# Iodine supplementation of lactating women and assessment of infant visual information processing and maternal and infant thyroid function: A randomized trial

**DOI:** 10.1371/journal.pone.0223348

**Published:** 2019-10-07

**Authors:** Tafere Gebreegziabher, Tesfaye Woltamo, David G. Thomas, Tay S. Kennedy, Barbara J. Stoecker

**Affiliations:** 1 Department of Health Sciences, Central Washington University, Ellensburg, WA, United States of America; 2 School of Environment, Gender, and Development Studies, Hawassa University, Hawassa, Ethiopia; 3 Department of Psychology, Oklahoma State University, Stillwater, OK, United States of America; 4 Department of Nutritional Sciences, Oklahoma State University, Stillwater, OK, United States of America; University of Ghana, GHANA

## Abstract

Iodine deficiency is one of the major causes of brain damage in childhood. However, iodine supplementation during early pregnancy and lactation can prevent the ill effects of iodine deficiency. This study evaluated maternal and infant thyroid function and infant visual information processing (VIP) in the context of maternal iodine supplementation. A community-based, randomized, supplementation trial was conducted. Mother infant dyads (n = 106) were enrolled within the first 10 days after delivery to participate in this study. Mothers were randomly assigned either to receive a potassium iodide capsule (225 μg iodine) daily for 26 weeks or iodized salt weekly for 26 weeks. Maternal thyroxine (T_4_), triiodothyronine (T_3_), thyroid stimulating hormone (TSH), thyroglobulin (Tg), urinary iodine concentration (UIC), breast milk iodine concentration (BMIC) and infant T_4_, TSH, UIC and VIP were measured as outcome variables. At baseline, neither mothers nor infants in the two groups were significantly different in any of the biomarkers or anthropometric measurements. Maternal TSH and goiter prevalence significantly decreased following iodine supplementation. The percentage of infants who preferentially remembered the familiar face was 26% in the capsule and 51% in the I-salt groups. Infant sex, length for age Z score, BMIC, maternal education and household food security were strong predictors of novelty quotient. In conclusion **s**upplementation daily for six months with an iodine capsule or the use of appropriately iodized salt for an equivalent time was sufficient to reduce goiter and TSH in lactating women. Higher BMIC and LAZ as well as better household food security, maternal education, and male sex predicted higher novelty quotient scores in the VIP paradigm.

## Introduction

Iodine is essential for the synthesis of thyroid hormones that regulate the metabolic processes of most cells and play important roles in human growth and development [1]. Iodine deficiency disorders (IDDs) are a major global public health problem and one of the most severe causes of preventable brain damage in childhood [2].

IDDs can be prevented and controlled by providing iodine and iodine can be provided in different ways. However, the United Nations Children’s Fund and the World Health Organization jointly recommended salt iodization where iodized salt is accessible. Iodized salt is a safe, cost effective and sustainable strategy to ensure sufficient intake of iodine by all individuals and to improve maternal and infant health [3–6]. Globally, over 70% of households use iodized salt [7]. In places where iodized salt is not accessible, the recommendation from WHO/UNICEF/ICCIDD has been a daily dose of 250 μg iodine supplement for pregnant and lactating women or a single annual dose of 400 mg iodine as an iodized oil supplement [4]. However, one recent study has reported that 150 μg of iodine daily was enough to reduce the number of women with insufficiency [8]. For children below 2 years of age, the recommendation is a daily dose of 90 μg iodine or a single annual dose of 200 mg iodine as an iodized oil supplement [4]. For infants 0 to 6 months of age, because a significant amount of iodine is secreted into breast milk, iodine should be available through breast milk, provided that the lactating mother is receiving sufficient iodine and her child is exclusively breastfed [9].

Although there are some studies that confirmed the effect of iodine supplement on thyroid status, thyroid gland and brain function [6, 10], little has been done to assess the efficacy of iodized salt in relation to these outcomes. In a prospective study in Denmark four years after a mandatory salt iodization program (13 mg/kg iodine), a lower median thyroid volume was observed in women aged 18 to 65 years. A larger relative decline of thyroid volume was observed in the younger compared to older females from a site where iodine deficiency was moderate [11]. In our study, women supplemented with adequately iodized salt had breast milk iodine concentrations that were not different from women who received a daily capsule containing 225 ug iodine; infant urinary iodine concentrations also were not significantly different based on source of maternal iodine supplement [12].

Ethiopia launched a national salt iodization program in 2012. However salt has been iodized manually using knapsack sprayers which makes it difficult to get a homogenous product. According to the Ethiopian Public Health Institute, of the 5605 salt samples tested nationally in 2014 only 42.7% had iodine content above 15 mg/kg, the rest varied between 0 and < 15 mg/kg [13].

The general objective of this study was to assess the efficacy of adequately iodized salt (30–40 mg/kg) for lactating women and for their breast-fed infants to the age of six months compared to a daily 225 μg iodine capsule. The specific objective of this study was to evaluate maternal and infant thyroid function and infant visual information processing (VIP) in the context of maternal iodine supplementation.

## Materials and methods

### Subjects and study design

The study was a community-based, randomized, supplementation trial conducted in 2013 in Sidama zone, southern Ethiopia. The study population depended on subsistence farming for their livelihood. Their major staple food was enset *(Enset ventricosum)* followed by unrefined maize [14]. The area was known for severe iodine deficiency prior to national implementation of salt iodization in 2012 [15–17]. Breast feeding behavior, household salt use, and compliance of subjects was reported in our previously published manuscript [12]. This study is registered with ClinicalTrials.gov, number NCT03889431.

### Subject selection methodology

This study enrolled all lactating women in the participating communities who delivered between January and February 2013. A total of 106 healthy mother-infant dyads were included in the study and data collection was completed by August 30, 2013. Eligibility criteria were: the women must be lactating and must have delivered a full-term single infant within the prior 10 days. Mothers and infants must have no history of illness, thyroid dysfunction or iodine supplementation. All mother-infant dyads who met the criteria were invited to volunteer for the study, and enrollment was continued until the required sample size was attained.

#### Ethical clearance

Ethical approval was obtained from Oklahoma State University (OSU), Hawassa University, the Ethiopian Ministry of Science and Technology, and the Food, Medicine, and Health Care Administration and Control Authority of Ethiopia. Following receipt of ethical clearance, study participants were given a detailed explanation of the objectives of the research and consent was obtained from the women for themselves and their infants before data collection was started. Women were enrolled with the help of health extension workers working in the study area.

#### Randomization

**Fig 1:**
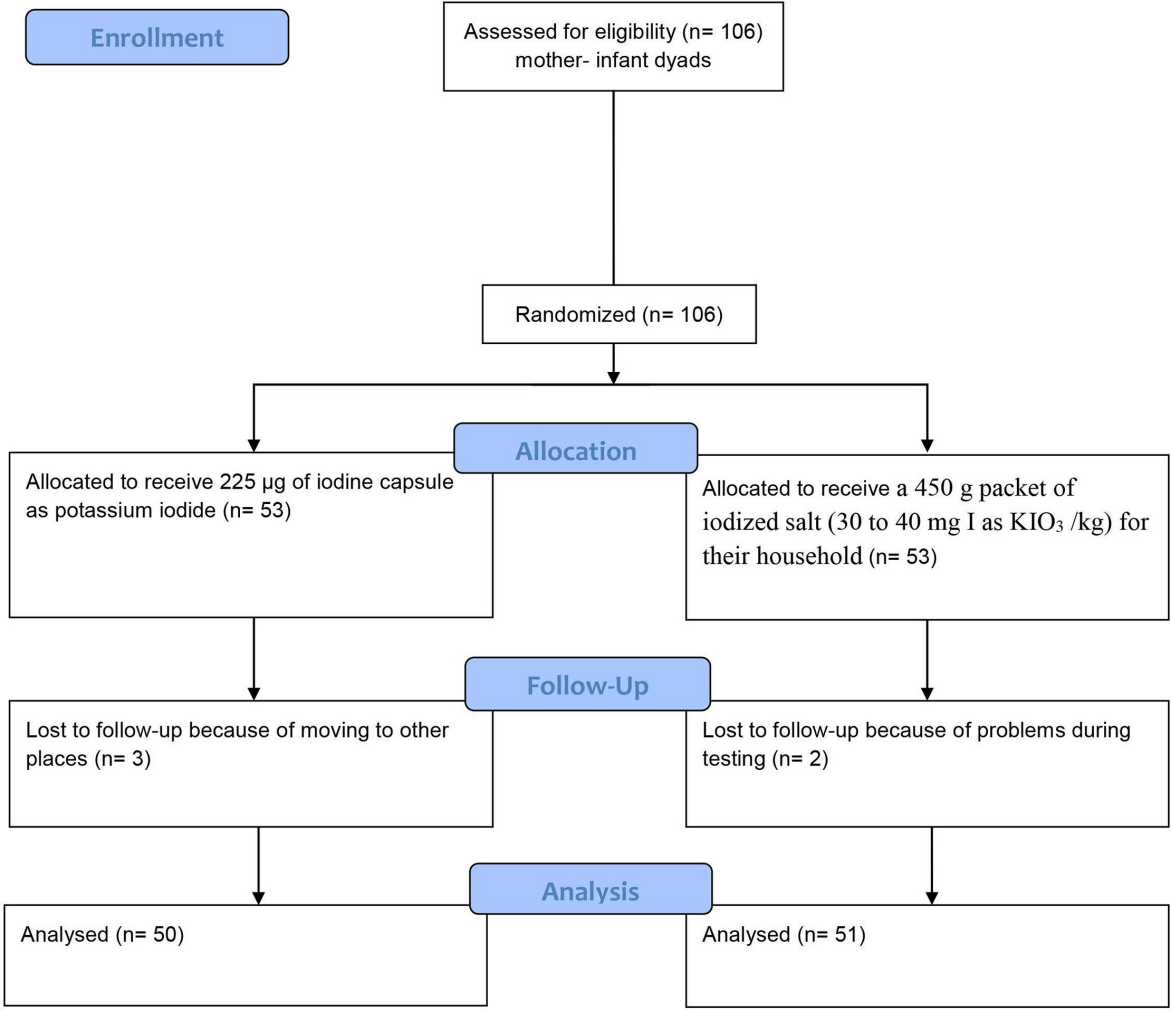
Research design. (CONSORT flow diagram).

The 106 mothers with their infants were randomly assigned either to the iodine capsule group or to the iodized salt (I-salt) group using random numbers (Fig 1).

#### Procedures

The capsule group (n = 53) received 225 μg of iodine daily as a capsule of potassium iodide (Pure Encapsulations, Inc., Boston, MA) for 26 weeks. Iodine capsules were ingested daily by mothers under supervision of the health extension worker. The I-salt group was provided weekly with a 450 g packet of iodized salt (30 to 40 mg I as KIO_3_ /kg) for their household for 26 weeks (Guts Agro Industry PLC, Addis Ababa, Ethiopia) by the health extension workers. The 450 g of salt provided weekly for each family was planned to be enough that they would not purchase any additional (perhaps inappropriately iodized) salt from the market. There was no requirement to use all of the provided salt.

A national survey conducted in 2005 with a multistage cluster sampling design estimated salt consumption per person per day using 24-hour dietary recalls. The national mean (±SD) for salt consumption was 8.4 (5.9) g/day. However, based on the 1,677 households sampled in the SNNPR region (our study region), salt consumption was 17.2 (13.8) g per person per day, or more than twice the national average. One reason for high salt consumption in the region is the custom for use of salt rather than sugar in coffee [18].

Prior to beginning supplementation, baseline data were collected from both mothers and infants. Collection and analysis of breast milk and urine has been described in our previous paper [12]. Maternal blood samples were collected using a disposable 10 cc syringe coated with lithium heparin with a 21 gauge needle (Sarstedt, Inc., Newton, N.C.). The blood was centrifuged and plasma was separated immediately. Plasma was frozen at– 20 ^0^C and analyzed for concentrations of triiodothyronine (T_3_), thyroxine (T_4_), thyroid stimulating hormone (TSH) and thyroglobulin (Tg).

Each woman was examined for goiter at baseline and end point by a single health officer using palpation based on the following grades: grade 0, no palpable or visible goiter; grade 1, palpable goiter but not visible when neck is in the normal position; grade 2, visible goiter when neck is in the normal position [4]. For mothers weight and height to calculate body mass index (BMI = Wt_kg_/Ht_m2_), and mid upper arm circumference (MUAC) were measured. Infant anthropometry including head circumference, weight and length were measured. Infants’ weight-for-age (WAZ), length-for-age (LAZ), weight-for-length (WLZ), and head circumference-for-age (HCAZ) were calculated using WHO Anthro software (Version 2.0.4). Anthropometric measurements were done in duplicate.

Mothers were interviewed at baseline using an individual questionnaire adapted from the Ethiopian Demographic and Health Survey. The questionnaire included socioeconomic status and demographic characteristics of the women [19]. Additionally, data were collected using the Household Food Insecurity Access Scale (HFIAS) developed by the Food and Nutrition Technical Assistance (FANTA) project of the United States Agency for International Development (USAID) [20].

After 26 weeks, collection of biological samples and anthropometric measurements were repeated. Additionally, at 26 weeks infant blood samples were collected on specimen paper by finger prick for TSH and T_4_ analysis, and the Visual Information Processing (VIP) test was administered to infants. All the participating mothers lactated throughout the study; thus no mother was excluded for having stopped lactation after enrollment.

### Administration of visual information processing (VIP) test

#### Materials

Testing of infant VIP was modified from an earlier report with Ethiopian infants [21] and from the work of Rose and colleagues [22]. For the VIP, eight pictures of young adult Ethiopian faces without emotional expression were used. One laptop computer controlled the presentation of stimuli. A second computer was used to follow and record the infant’s looking behavior. The infant’s looking behavior was coded live, but a camera in the second computer recorded the VIP test for later reliability testing. VIP measurements were always conducted by the same person.

#### VIP test procedures

For the VIP test, the infant sat on the mother’s lap half a meter from a 15” laptop screen in a darkened room at the health center. The tester (who was blind to the study and out of sight of the infant) focused the camera in the second laptop on the infant’s eyes. The habituation phase began with the presentation of one face randomly selected from the eight faces. When the infant looked at a picture, its reflection was visible in the infant’s pupil and the experimenter pressed a key on his computer. Response latency (time between stimulus onset and the look) and the duration of the look were recorded by the computer software. When the infant looked away, the experimenter released the key; if the look away lasted 1s or longer, the face disappeared from the screen for 2s and then reappeared. This procedure continued until the criterion for habituation was achieved, namely two consecutive looks that were less than one half the mean of the two longest looks.

After habituation, the comparison phase began without interruption. In the comparison phase, the face that appeared during habituation (the “familiar” face) appeared again on one side of the screen and a novel face of the opposite sex appeared on the other side of the screen. The faces remained on the screen regardless of whether or not the infant was looking at one of them. Once the infant accrued a total looking time at the faces of 5s, the faces disappeared for 1s, and then reappeared but on opposite sides of the screen. They remained until an additional 5s of looking had accrued. The infant’s look duration, shifts between faces, and looks away from the pictures were compiled simultaneously by the software.

Visual recognition memory is composed of various constructs [22]. During habituation, look duration measures processing speed and attention [23] and average look duration and number of looks reflect the speed at which recognition was achieved [24]. For the comparison phase, the novelty quotient (NQ; preference for the new face) measures recognition memory (21) and total shifts identify how active the infant was in comparing the two faces.

A university student, blind to the study, coded recorded videos for 30 randomly selected infants to test coding reliability. For longest look, the correlation was 0.78; correlation for novelty preference was low probably because distinguishing between infant’s looks at the right and left side of the screen was more difficult in the taped version than in person.

### Measurement of biomarkers

The T_3_, T_4_, TSH and Tg were quantitatively determined by ELISA assay (ALPCO Diagnostics, Salem, NH). Quality control for T_3_ and T_4_ were obtained from Bio-Rad Laboratories (Irvine, Ca). Infant’s T_4_ and TSH also were determined by ELISA assay (Diagnostic Automation, Calabasas, CA). Reference ranges for thyroid hormone biomarkers of mothers and infants are included as supplied by the manufacturers. Hence, for mothers TSH: 0.4–4.2 μIU/mL, T_3_: 0.75–2.2 ng/mL, T_4_: 5–13 μg/dL and Tg: 2–50 ng/mL. Reference ranges for infants were: TSH: 0.7–34 μIU/mL and T_4:_ 8–23 μg/dL.

### Statistical analysis

Percentages, frequency distributions, means (SD) and medians (IQR) were used to describe the socio-economic status, demographic characteristics, and iodine status of lactating mothers and their infants. All data were checked for normal distribution and skewed data were log-transformed before analysis. To compare the means between the two treatment groups independent-sample *t*-tests were used for normally distributed data and the Mann-Whitney U test for non-normally distributed data. For comparison of samples at baseline and 26 weeks, paired-sample *t*-tests were used for normally distributed data and Wilcoxon signed-Rank tests were used for non-normally distributed data.

Pearson’s correlation coefficient (*r*) or Spearman’s rho were used to examine relations between variables. Variables predicting VIP were analyzed by multiple regression. All variables were checked for collinearity using variance inflation factors (VIF). All of the biomarkers analyzed and infants’ anthropometric indices with VIF < 4 were entered into the models.

Multiple classification analysis (MCA) was used for predicting novelty quotient (NQ). MCA examines the contribution of each category of the predetermined predictor variables before and after adjustment for the control variables. MCA is effective to control multicollinearity [25]. Post hoc power calculation was made using STATA software for four outcomes (Tg, T_3_, T_4_ and TSH) and the power was adequate (>80%) for the Tg and TSH outcomes. However, it was lower than 80% for T_3_ and T_4_.

## Results

A total of 106 mother-infant pairs were recruited between Jan and Feb 10, 2013 and data collection was completed by Aug 30, 2013. Three mother-infant pairs were lost from the capsule group because of moving to other places and two mother-infant pairs were lost from the I-salt group because of problems during testing. The baseline characteristics of treatment groups are presented in Table 1. There were no significant differences between groups in any measured variables for mothers or infants at baseline. The median maternal TSH, T_3_, T_4_ and Tg were all within the reference ranges. Visible goiter was seen in 52% of women at baseline and an additional 23% had palpable goiter.

**Table 1:** Socio-demographic, thyroid hormones and goiter of mothers and anthropometry of infants at baseline and at 26 weeks.

	Baseline			26 weeks		
	Capsule group (n = 50)	I-salt group (n = 51)	p—value	Capsule group (n = 50)	I-salt group (n = 51)	p—value
Mothers	Mean (SD), median (IQR) or n (%)	Mean (SD), median (IQR) or n (%)		Mean (SD), median (IQR) or n (%)	Mean (SD), median (IQR) or n (%)	
- Age (years)	23 (20, 27)	21 (20, 25)	0.14			
- MUAC (cm)	23.2 (1.8)	23.3 (1.7)	0.62			
- BMI (kg/m^2^)	21.2 (2.5)	21.8 (2.0)	0.15			
- Gravidity	2 (1.75, 4)	2 (1, 5)	0.41			
- Parity	2 (1, 4)	2 (1, 4)	0.38			
- School years	2 (2, 3)	2 (1, 3)	0.42			
- T_3_ (ng/mL)	1.3 (0.3)	1.4 (0.3)	0.06	1.14 (0.4)	1.25 (0.4)	0.17
- T_4_ (μg/dL)	6.7 (5.9, 7.2)	6.8 (5.4, 7.2)	0.96	6.7 (5.5, 8)	5.4 (4.8, 6.9)	0.02
- Tg (ng/mL)	4.2 (1.6, 9.3)	4.2 (2.3, 9.5)	0.73	4.2 (1.8, 9)	4.2 (2, 8)	0.61
- TSH (μIU/mL)	2.6 (1.1, 4.4)	1.9 (0.8, 3.2)	0.29	1.2 (0.9, 2.4)	1.1 (0.7, 1.8)	0.16
- Goiter			0.96			0.14
- Grade 0	12 (24%)	13 (26%)		35 (70%)	29 (57%)	
- Grade 1	11 (22%)	12 (23%)		15 (30%)	21 (41%)	
- Grade 2	27 (54%)	26 (51%)		0	1 (2%)	
Infants						
- Age (days)	5 (3, 7)	6 (5, 8)	0.06			
- WAZ	-0.29 (1.23)	-0.43 (1.37)	0.59	-0.35 (1.3)	-0.4 (1.1)	0.82
- WLZ	-0.38 (1.9)	-0.44 (1.14)	0.80	0.38 (1.1)	0.46 (1.2)	0.72
- LAZ	-0.28 (1.44)	-0.41 (1.47)	0.66	-0.8 (1.3)	-1.1 (1.1)	0.35
- HCAZ	1.45 (0.4, 1.9)	1.09 (0.1, 1.7)	0.16	0.73 (0.24, 1.62)	0.78 (0.24, 1.60)	0.34

Data are median, mean or percentage. Continuous data were analyzed using T-Test and Mann-Whitney U test, categorical variable was analyzed using Chi-square test and normality was checked using Kolmogorov-Smirnov. T_3_ = triiodothyronine, T_4_ = Thyroxin, Tg = thyroglobulin, TSH = thyroid stimulating hormone, MUAC = mid-upper-circumference, BMI = body mass index

The median mothers’ age was 22 (20, 25) years, mean body mass index (BMI) was 21.5 (2.3) and mid-upper-arm-circumference (MUAC) was 23.3 (1.7) cm. The median infant age was 5 (3, 7) days. Excluding the five subjects who dropped out the study, there were 50 (49.9%) male and 51 (50.5%) female infants participating in the study. Infants did not differ in any of the anthropometric indices.

As shown in Table 1, there were no significant differences between mothers in the capsule group and I-salt group for TSH, T_3_ and Tg at 26 weeks, but T_4_ was significantly lower in the I-salt group. Only T_4_ (p = 0.014) showed a treatment by time interaction. Combined prevalence of visible and palpable goiter at 26 weeks tended to be higher in the I-salt group although not significant (p = 0.14).

Infants’ TSH and T_4_ at 26 weeks were within reference ranges in both groups (Table 2). However unlike mothers, infants’ T_4_ was higher in the I-salt group. Similar to baseline, infants were not significantly different in any of the anthropometry indices at 26 weeks (Table 1).

**Table 2:** Thyroid hormones and visual information processing variables of infants at 26 weeks.

	Capsule group (50)	I-salt group (51)	p—value
TSH (μIU/mL)	0.56 (0.1, 1.6)	0.51 (0.1, 1.3)	0.92
T_4_ (μg/dL)	10.8 (8.7, 13.6)	13.9 (10.6, 17.6)	0.003
VIP variables of infants			
Total shift	4 (2, 6)	4 (2, 6)	0.92
No. of looks	6 (5, 7)	6 (5, 8)	0.09
Average look	9.6 (5.8)	8.0 (5.2)	0.18
Longest look	22.5 (17.8)	19.5 (19.1)	0.21
Total novelty quotient (> 0.55)			0.02
- Yes	13 (26%)	26 (51%)	
- No	37 (74%)	25 (49%)	

In the VIP tests infants were different (p < 0.02) by treatment for novelty quotient but not for the other VIP measures. A novelty quotient > 0.55 indicates that the infant remembers the familiar face [22, 23]. Based on this classification, 26% of the infants in the capsule and 51% in the I-salt group remembered the familiar face (Table 2).

**Fig 2:**
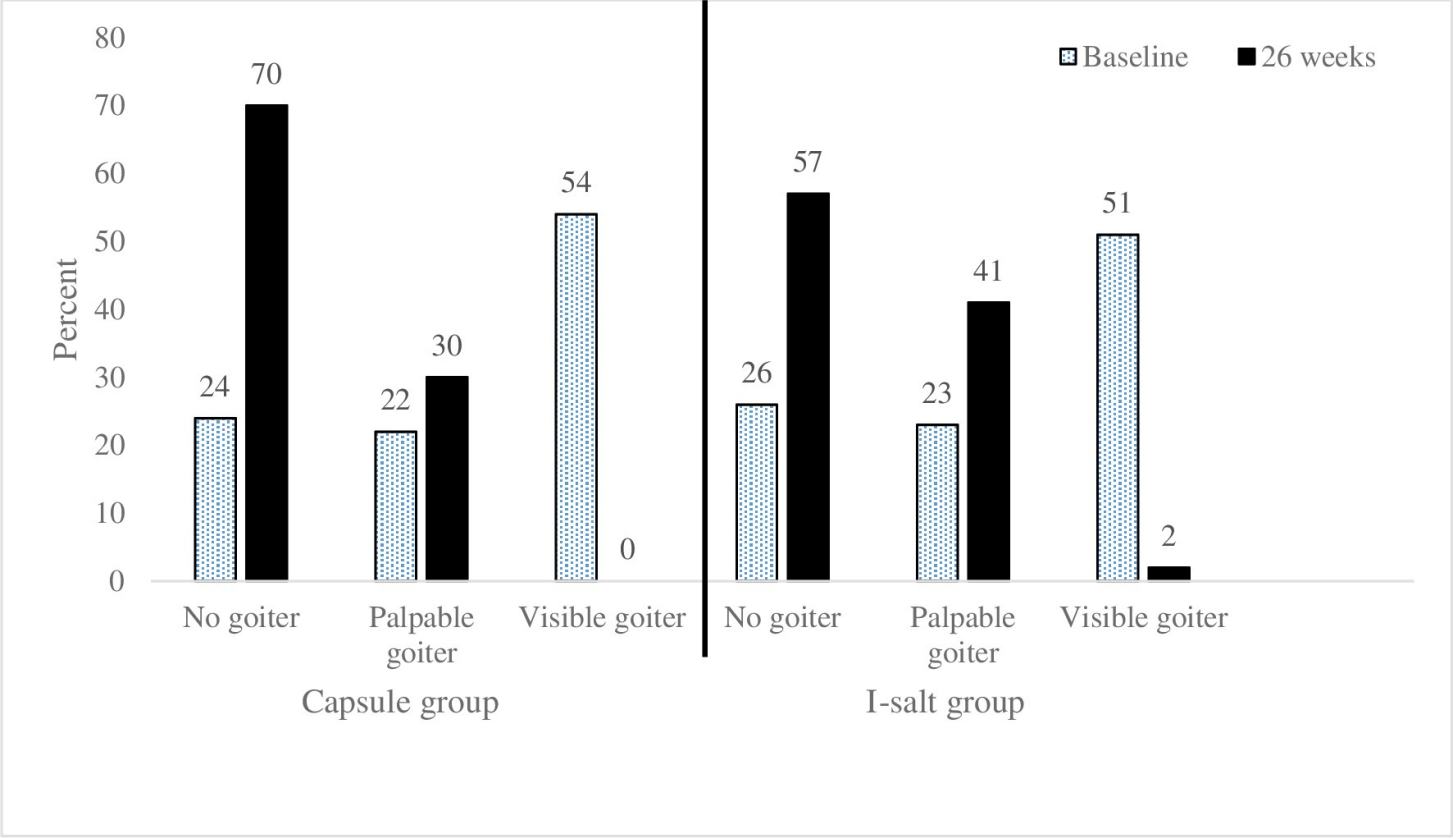
Prevalence of goiter in women at baseline and 26 weeks in the capsule vs salt groups (n = 101).

As shown in Fig 2, maternal goiter decreased from 76% at baseline to 30% at 26 weeks (X^2^ = 7.9, p = 0.02) in the capsule group and from 74% at baseline to 43% at 26 weeks (X^2^ = 21.1, p < 0.001) in the I-salt group. Maternal TSH was associated with goiter (X^2^ = 5.8, p = 0.016) at baseline but not at 26 weeks when all TSH values were much lower.

Because there were few significant differences between groups at 26 weeks based on iodine source, data from both supplementation groups were combined to examine effects of either source of iodine in lactating women and their infants (Table 3). For the group as a whole, the median maternal TSH and prevalence of maternal goiter decreased significantly after 26 weeks of access to iodine. Total goiter declined from 76% at baseline to 37% at 26 weeks (p < 0.001).

**Table 3:** Thyroid hormone biomarkers of mothers and anthropometry of infants at baseline and at 26 weeks in participants who received either iodine capsule or iodized salt (n = 101).

	Baseline	26 weeks	p- value
Mothers			
TSH (μIU/mL)	1.9 (0.8, 3.7)	1.2 (0.7, 2.1)	0.001
T_4_ (μg/dL)	6.8 (5.7, 7)	5.8 (5.1, 7.5)	0.14
T_3_ (ng/mL)	1.33 (0.3)	1.27(0.3)	0.19
Tg (ng/mL)	4.2 (2.3, 9.5)	4.2 (2, 7.1)	0.266
Goiter			< 0.001
- Grade 0	25 (24.7%)	64 (63.4%)	
- Grade 1	23 (22.8%)	36 (35.6%)	
- Grade 2	53 (52.5%)	1 (1.0%)	
Infants			
WAZ	-0.34 (-0.9, 0.4)	-0.4 (-0.9, 0.4)	0.91
WLZ	-0.4 (-1.0, 0.5)	0.4 (-0.4, 1.2)	< 0.001
LAZ	-0.3 (-1.4, 0.7)	-0.9 (-1.7, -0.1)	0.006
HACZ	1.3 (0.2, 1.9)	0.7 (0.2, 1.6)	0.88

Data are median (IQR), mean (SD) and percentages. Continuous data are analyzed using paired samples T-test or Wilcoxon, proportions (percentages) were analyzed using Chi-square test.

Infants in the combined groups (Table 3) did not differ in WAZ and HCAZ but there were significant differences in WLZ and LAZ at 26 weeks compared to baseline. The means of these anthropometric Z-scores were above the cutoff level set for nutritional insult (≤ -2 Z score); however, 14% were stunted, 13% were underweight and 8% were wasted at baseline and at 26 weeks, 18% were stunted, 11% were underweight and 3% were wasted. Males had significantly better scores than females for total novelty quotient (X^2^ = 4.54, p = 0.033) and number of looks (X^2^ = 6.6, p = 0.011).

Table 4 shows significant predictors of the VIP variables using stepwise multiple linear regression analysis. One unit increase in infant urinary iodine adjusted for creatinine at 26 weeks predicted 0.24 more total shifts by the infant. Maternal T_3_ at 26 weeks predicted increased total shifts of 0.21 units and maternal T_4_ at baseline predicted increased total shifts of 0.23 units. About 12% of the variation in total shift was explained by the model.

**Table 4:** Multiple regression predicting VIP tests using stepwise criteria (n = 101).

Dependent variables	Predictors	Beta	p
Total number of shifts	Infant UIC/Cre (μg/L)at 26 weeks	0.24	0.02
	Maternal T_3_ (ng/mL) at 26 weeks	0.21	0.043
	Maternal T_4_ (μg/dL) at baseline	0.23	0.029
	Adjusted R square = 0.12		
Average look	Maternal T_3_ (ng/mL) at baseline	-0.26	0.008
	Maternal Tg (ng/mL) at baseline	-0.24	0.015
	Adjusted R square = 0.104		
Longest look	Maternal Tg (ng/mL) at baseline	-0.25	0.012
	Maternal T_3_ (ng/mL) at baseline	-0.24	0.016
	Adjusted R square = 0.10		

A one unit increase in maternal T_3_ at baseline predicted a 0.26 unit decrease in average look and one unit increase in Tg at baseline was associated with a 0.24 unit decrease in average look. The model explained 10.4% of the variation in average look.

For longest look, one unit increase in maternal Tg at baseline was associated with a 0.25 unit decrease in longest look and one unit higher maternal T_3_ at baseline was associated with a 0.24 unit decrease in longest look; 10% of the variation in longest look was explained by the model.

Multiple classification analysis results (Table 5) showed that several variables significantly predicted infants NQ including sex (p = .027), LAZ (p = .015), BMIC (p = .042), maternal education (p = .004) and HFIAS score (p = .042). Taking the beta (β) coefficients as indicators, household food security, women education and LAZ were strongest predictors compared with the other four predictors included in the model. Infants from food secure households had significantly higher NQ (0.638) compared to infants from food insecure households and infants from literate mothers had significantly higher NQ (0.55) compared to infants from illiterate mothers. Likewise, infants whose LAZ score was above -1 SD had significantly higher NQ (0.55) compared to infants with lower LAZ scores. Moreover, infants whose mothers had BMIC ≥ 120 μg/L and infants who were male had significantly higher NQ than their counterparts.

**Table 5:** Result of multiple classification analysis (MCA) for the key determinants of infants novelty quotient NQ by selected predictors and covariates (n = 101).

Variable	Mean total NQ					
	n	Unadjusted mean	Eta (η)	Adjusted mean	Beta (β)	Sig
Child sex			.214		.205	.027^b^
Male	50	.548		.546		
Female	51	.468		.476		
LAZ score			.207		.240	.015^b^
< -1 SD	48	.471		.465		
≥ -1 SD	53	.548		.554		
WAZ score			.046		.173	.274
< -1 SD	24	.523		.450		
≥ -1 SD	77	.503		.525		
Infant T_4_ (μg/dL)			.15		.06	.326
< 8.0	9	.56		.546		
> 8–23	92	.51		.504		
BMIC (μg/L)*			.196		.211	.042^b^
< 120	55	.487		.485		
≥ 120	46	.571		.560		
Women’s education			.207		.277	.004^a^
Illiterate	38	.458		.441		
Literate	63	.537		.548		
HFIAS score**			.260		.339	.042^b^
Food secure	58	.621		.638		
Mild food insecurity	12	.559		.605		
Moderate food insecurity	20	.486		.480		
Severe food insecurity	11	.472		.472		

r = 0.59; R^2^ = 0.331; grand mean = 0.51

^a^ Significant α .01

^b^ Significant α .05

*BMIC–Breast milk iodine concentration

**HFIAS–Household food insecurity access scale

## Discussion

We examined the effects of 26 weeks of iodine supplementation on thyroid hormones, thyroglobulin and goiter of lactating women and on T_4_, TSH and VIP in infants. At baseline mothers and infants in the two supplemented groups (capsule and I-salt) were not significantly different in any of the biomarkers or other characteristics.

The study population had been known to have severe iodine deficiency for decades [15–18, 26]. The current study was conducted in 2013, following the rapid implementation of a salt iodization program in 2012. Presumably as the result of the program implementation, our study population showed higher UIC and BMIC than previous studies [12].

Following 26 weeks of iodine supplementation, plasma TSH in lactating women significantly decreased by 54% in the capsule group and by 42% in the I-salt group compared to baseline. However, these improvements in TSH were not significantly different between the two groups of mothers. Santiago and colleagues reported there was no significant difference in maternal TSH between pregnant women who were supplemented with iodized salt and those who were supplemented with either 200 μg iodide per day or 300 μg iodide per day [27]. A study that compared a control group to women who received 300 μg iodine as potassium iodide during pregnancy and lactation reported that plasma T_4_ and T_3_ were significantly higher in the supplemented women; TSH was significantly higher in the control group [28].

Because a majority of a breast feeding mother’s iodine intake is secreted into the breast milk, an exclusively breastfed infant may have better iodine status than the mother [29]. Most of the infants in the current study had normal T_4_ and all had TSH within the normal range.

Thyroid hormones did not respond consistently to iodine supplementation in this study. Maternal TSH improved significantly following iodine supplementation, T_3_ and T_4_ showed decreased values after supplementation but the changes were not significant. The demands for iodine during lactation may reduce thyroid hormones if iodine intakes are marginal. However, at baseline 90% of the women had T_3_ and 82% had T_4_ within the reference range; values were quite similar at 26 weeks when 86% of women had T_3_ and 77% had T_4_ within the reference range and there was no change in Tg.

In a double-blind, randomized, controlled trial conducted in Morocco, a single capsule of 400 mg of iodine as iodized oil given to the breast-feeding mother or a single oral supplement of 100 mg of iodine given to the newborn baby did not produce a significant change in maternal or infant TSH or T_4_ at 3 months, 6 months or 9 months after supplementation [30]. Such inconsistent thyroid hormone response to iodine supplement was also reported in other studies [28, 31]. For example, pregnant women showed no change or an increase in maternal TSH following iodine supplementation of 50 μg daily, 200 μg daily or 300 μg daily for six months [27, 32]. In two other randomized controlled trials, maternal free T_4_ concentration decreased in both controls and treated groups and no difference was observed between groups [33].

The inconsistency of thyroid hormone response to iodine supplement could be because circulating thyroid hormones depend on iodine deficiency status, on increased needs during pregnancy and lactation and on age. A recently published review on biomarkers of iodine status suggested that T_4_ and T_3_ are good indicators of iodine status in areas of severe iodine deficiency and TSH was considered a sensitive marker in newborns [34]. Thyroglobulin has been recommended as a more sensitive indicator than TSH and T_4_ in iodine deficient and excess areas in children because thyroglobulin is synthesized only in the thyroid [34, 35]. Another group commented that goiter rate, Tg and TSH are good markers to assess iodine status in a population but thyroid hormones are fairly imprecise to detect iodine deficiency [36]. However, unlike for children, no cutoff point for Tg for adults has been established related to iodine status [35]. In one study, serum Tg was correlated with severity of iodine deficiency assessed by urinary iodine [34]. In the current study Tg didn’t show any correlation with urinary iodine. When thyroid markers were categorized to examine association with goiter rate, TSH at baseline was the only variable that showed significant association with goiter (X^2^ = 5.8, p = 0.016).

In the present study maternal goiter significantly decreased in both capsule and I-salt groups despite six months of lactation. In goitrous children age 6 to 12 years in CÔte d’Ivoire, a 200 mg iodine supplement produced a significant and continuous decrease in thyroid gland volume over 30 weeks. Similar to our results there was no significant change in serum T_4,_ but TSH decreased significantly in the CÔte d’Ivoire study [37]. In contrast, in a severely iodine deficient area in Senegal, 480 mg of iodine given as iodized oil to adults significantly increased T_4_ and decreased rate of goiter, T_3_ and TSH [38].

The effect of iodine supplement on maternal thyroid disorders and in children’s neurocognitive development in severely iodine deficient areas is well established [39]. It is unclear however, the extent to which maternal iodine supplementation in areas of mild-to-moderate iodine deficiency may affect cognitive function of their offspring [10, 40]. Children of pregnant women who had received iodine supplementation from their first trimester compared with children of unsupplemented women showed no difference in mental development index measured by the Bayley Scales of Infant Development (BSID) at the age of two years [28]. However, the children of supplemented mothers had higher scores on the psychomotor development index of the BSID. On the other hand, a prospective study reported that children of mothers exposed to mild-moderate iodine deficiency showed attention deficit and hyperactivity disorders [41].

In our study, the look duration (length of the longest look) during the familiarization phase (habituation phase) as a measure of attention and processing speed was not significantly different between groups. The mean of the longest look duration in both groups was 21.1 (18.0)s. Shorter fixation indicates better attention and faster processing speed and is a predictor of later childhood IQ [23, 42]. In the current study more than 70% of the infants in both groups were below the mean with 26% below 10s. The average look duration, which indicates the speed at which recognition of the familiar stimulus is achieved [24], likewise was not significantly different between groups.

Although there is no established standard for number of looks a child would make during the habituation phase or trials to criterion, infants were not significantly different between groups. However, the longer it takes an infant to reach the habituation criterion (i.e., the greater the number of looks before criterion is reached), the longer it is taking the infant to learn the stimulus. In this regard 43% of the infants had number of looks above the median and 35% had number of looks below the median of 6 (5, 7.7); those with few looks learned the habituation face more quickly [43]. In total (both groups combined) the mean NQ was 0.51 (0.19) which is similar with the mean novelty preference reported by Oakes and Kovack-Lesh [44] and is higher than the mean NQ reported by Kennedy and colleagues eight years earlier (before introduction of iodized salt) in infants 6–8 months age in a similar area in Ethiopia [21].

The VIP paradigm has been used to assess infants’ and children’s information processing speed, recognition memory and attention in relation to various factors. Infants whose mothers had high blood DHA concentration showed faster information processing speed over the first year as well as less distractibility in the second year than those whose mothers had low blood DHA [45]. A study conducted in Ethiopian infants showed that growth was associated with increased performance in the VIP [21] paradigm. In another study, increased prenatal alcohol exposure was correlated with longer looks, indicating slower information processing [46]. Similarly, exposure to toxic substances during pregnancy resulted in poorer recognition memory during the first year of the child’s life [47].

In the present study we have examined possible associations and predictor variables of VIP of infants. Accordingly, NQ was affected by infants’ TSH, T_4_, anthropometry indices, maternal BMIC and education as well as household food security state. Moreover, male infants remembered the familiar face and learned faster than female infants. Not much evidence is available regarding iodine intake on infant cognition performance by sex. One study done in 1982 in school children from an area of endemic goiter reported that girls showed better cognition improvement as a result of iodine supplement than boys [48]. Another study from iodine sufficient and mildly iodine deficient areas reported similar results [49]. However in this study, because male and female infants didn’t differ in either UIC, T_4_ or TSH, the difference observed in NQ could be independent of iodine intake. For instance, it could be related to amount of breast milk consumed or infant stimulation by caretakers [50, 51].

Based on the multiple regression analyses maternal thyroid hormones at baseline were significant predictors of some of the VIP variables. Concentrations one week after delivery may reflect the maternal thyroid hormone status during pregnancy which might have affected the child’s brain development. However, none of the VIP variables were significantly different by maternal goiter grade at baseline. Moreover, comparison of thyroid biomarkers and VIP data for mothers with UIC above and below 100 ug/L were not significantly different.

We performed reliability checks for longest look and novelty preference. The correlation for longest look was 0.78. However, correlations for novelty preference were lower. This poor reliability was likely due to the quality of the recorded video and the fact that the stimuli were presented on a single laptop screen, which made it difficult to tell on the recorded video whether the child was looking at one of the pictures or farther to the side and thus away from the pictures. For the future this problem could be solved by having a better video recording and/or by using two laptop screens placed farther apart.

A limitation of this study was the relatively small sample size which was calculated based on outcomes for the original study [12]. Based on the post-hoc power calculation, the null association between the intervention and T_3_ and T_4_ may be due to insufficient power.

In conclusion, novelty quotient of infants was predicted in the VIP paradigm by higher maternal education, BMIC and LAZ, as well as by better household food security, and male sex. Attention to iodine status and other modifiable factors has the potential to impact optimal child development.

Although the study population had a long history of iodine deficiency, salt iodization had been implemented during the previous year. The present study found a median UIC at baseline that indicated community level iodine sufficiency based on WHO standards with median UIC less than 100 μg/L classified as iodine deficient. The median UIC at baseline for the mothers was 107 (71, 161) μg/L and for the infants was 218 (108, 356) μg/L. Nonetheless, on the basis of UIC, 46% of the women and 21% of the infants would be classified as at least mildly iodine deficient at baseline [4]. But effects of maternal iodine supplementation on thyroid function may have been muted because nearly half of the women were iodine sufficient. Biomarkers measured in this study would not be expected to be as responsive to iodine supplementation in iodine sufficiency. In the present study, only TSH and goiter showed improvement with supplementation in women and the only biomarker that showed association with goiter was TSH. Further investigation of TSH as a biomarker for assessment of iodine nutrition even in adults may be warranted. Moreover, because several minerals including iron and selenium play essential roles in thyroid hormone metabolism, it is important to assess the impact of these nutrients in both mothers and their infants [52].

This study confirmed that iodized salt showed similar effects on iodine status and thyroid hormones as a daily iodine capsule supplement if the salt is adequately iodized and closely monitored. Data for the present study were collected within a year after the salt iodization program was launched in Ethiopia, so it is too early to comment on the long term effects of an adequate supply of iodine to the population or on the quality control of the iodization process. Large scale and repeated monitoring studies likely are warranted in the interest of optimizing iodine status of the Ethiopian population.

## Supporting information

S1 File Study protocol. (PDF)

S2 File CONSORT checklist. (DOC)

S1 Dataset Data. (PDF)

## References

[1] Chan S, Kilby MD. Thyroid hormone and central nervous system development. J Endocrinol. 2000; 165:1–8. 10.1677/joe.0.1650001 10750030

[2] WHO: Iodine status worldwide. WHO global database on iodine deficiency Genenva: WHO 2004:1–58 (www.whqlibdoc.who.int/publication/2004/9241592001.pdf).

[3] Zimmermann MB. The effects of iodine deficiency in pregnancy and infancy. Paediatr Perinat Epidemiol. 2012; 26:108–117. 10.1111/j.1365-3016.2012.01275.x 22742605

[4] WHO/UNICEF/ICCIDD: Assessment of iodine deficiency disorders and monitoring their elimination a guide for program managers. Third edition World Health Organization 2007.

[5] Dold S, Zimmermann MB, Jukic T, Kusic Z, Jia Q, Sang Z, et al Universal salt iodization provides sufficient dietary iodine to achieve adequate iodine nutrition during the first 1000 days: A cross-sectional multicenter study. J Nutr. 2018; 148(4):587–598. 10.1093/jn/nxy015 29659964

[6] Robinson SM, Crozier SR, Miles EA, Gale CR, Calder PC, Cooper C, et al Preconception maternal iodine status is positively associated with IQ but not with measures of executive function in childhood. J Nutr. 2018; 148:959–966. 10.1093/jn/nxy054 29767745 PMC5991217

[7] Pearce EN, Andersson M, Zimmermann MB. Global iodine nutrition: Where do we stand in 2013? Thyroid. 2013; 23(5):523–528. 10.1089/thy.2013.0128 23472655

[8] Vongchana M, Ounjaijean S, Tongsong T, Traisrisilp K. The effectiveness of iodine supplementation during pregnancies in geographical areas of high prevalence of iodine insufficiency. J Obstet Gynaecol. 2018; 38(6):756–761. 10.1080/01443615.2017.1410534 29526129

[9] Andersson M, de Benoist B, Delange F, Zupan J. Prevention and control of iodine deficiency in pregnant and lactating women and in children less than 2-years-old: conclusions and recommendations of the Technical Consultation. Public Health Nutr. 2007; 10(12A):1606–1611. 10.1017/S1368980007361004 18053287

[10] Zimmermann MB. Iodine deficiency in pregnancy and the effects of maternal iodine supplementation on the offspring: a review. Am J Clin Nutr. 2009; 89 (suppl):668S–672S. 19088150 10.3945/ajcn.2008.26811C

[11] Vejbjerg P, Knudsen N, Perrild H, Carle A, Laurberg P, Pedersen IB, et al Effect of a mandatory iodization program on thyroid gland volume based on individuals' age, gender, and preceding severity of dietary iodine deficiency: a prospective, population-based study. J Clin Endocrinol Metab. 2007; 92(4):1397–1401. 10.1210/jc.2006-2580 17264188

[12] Gebreegziabher T, Stoecker BJ. Comparison of two sources of iodine delivery on breast milk iodine and maternal and infant urinary iodine concentrations in southern Ethiopia: A randomized trial. Food Sci Nutr. 2017; 5(4):921–928. 10.1002/fsn3.477 28748081 PMC5520864

[13] Ethiopian Public Health Institute: National salt iodization coverage towards prevention of iodine deficiency disorders in Ethiopia. 2014.

[14] Abebe Y, Bogale A, Hambidge KM, Stoecker BJ, Bailey K, Gibson RS. Phytate, zinc, iron and calcium content of selected raw and prepared foods consumed in rural Sidama, Southern Ethiopia, and implications for bioavailability. J Food Comp Anal. 2007; 20:161–168.

[15] Bogale A, Abebe Y, Stoecker BJ, Abuye C, Ketema K, Hambidge KM. Iodine status and cognitive function of women and their five year-old children in rural Sidama, southern Ethiopia. East Afr J Public Health. 2009; 6:296–299. 20803922

[16] Ersino G, Tadele H, Bogale A, Abuye C, Stoecker BJ. Clinical assessment of goiter and low urinary iodine concentration depict presence of severe iodine deficiency in pregnant Ethiopian women: a cross-sectional study in rural Sidama, southern Ethiopia. Ethiop Med J. 2013; 51(2):133–141. 24079157

[17] Gebreegziabher T, Teyike N, Mulugeta A, Abebe Y, Hambidge KM, Stoecker BJ. Lack of dietary sources of iodine and the prevalence of iodine deficiency in rural women from Sidama zone, southern Ethiopia. Afr J Food Agric Nutr Dev. 2013; 13:8401–8414.

[18] Abuye C, Berhane Y, Akalu G, Getahun Z, Ersumo T. Prevalence of goiter in children 6 to 12 years of age in Ethiopia. Food Nutr Bull. 2007; 28:391–397. 10.1177/156482650702800403 18274165

[19] Central Statistical Agency and ORC Macro: Ethiopian demographic and health survey. Central Statistical Agency Addis Ababa, Ethiopia. 2005.

[20] Coates J, Swindale A, Blinsky P: Household food insecurity access scale (HFIAS) for measurement of household food access: Indicator guide (v.3) Washington D.C: Food and Nutrition Technical Assistance Project, Academy for Educational Development 2007.

[21] Kennedy T, Thomas DG, Woltamo T, Abebe Y, Hubbs-Tait L, Sykova S, et al Growth and visual information processing in infants in Southern Ethiopia. J Appl Dev Psychol. 2008; 29(2):129–140. 10.1016/j.appdev.2007.12.003 19684873 PMC2726747

[22] Rose SA, Feldman JF, Jankowski JJ. Infant visual recognition memory. Dev Rev. 2004; 24:74–100. 10.1037/0012-1649.39.3.563 12760523

[23] Colombo J, Shaddy JD, Richman AW, Maikranz MJ, Blaga MO. The developmental course of habituation in infancy and preschool outcome. Infancy 2004; 5:1–38.

[24] Courage LM, Howe LM, Squires ES. Individual differences in 3.5-month-olds' visual attention: what do they predict at 1 year? Infant Behav Dev. 2004; 27:19–30.

[25] Susel A: Multiple classification analysis: Theory and application to demography Department of quanatitative methods in management, Nowy Sacz School of Business-National Luise University 2011.

[26] Girma M, Loha E, Bogale A, Teyike N, Abuye C, Stoecker BJ. Iodine deficiency in primary school children and knowledge of iodine deficiency and iodized salt among caretakers in Hawassa town: Southern Ethiopia. Ethiop J Health Dev. 2012; 26:30–35.

[27] Santiago P, Velasco I, Muela JA, Sanchez B, Martinez J, Rodriguez A, et al Infant neurocognitive development is independent of the use of iodised salt or iodine supplements given during pregnancy. Br J Nutr. 2013; 110(5):831–839. 10.1017/S0007114512005880 23375074

[28] Velasco I, Carreira M, Santiago P, Muela JA, Garcia-Fuentes E, Sanchez-Munoz B, et al Effect of iodine prophylaxis during pregnancy on neurocognitive development of children during the first two years of life. J Clin Endocrinol Metab. 2009, 94(9):3234–3241. 10.1210/jc.2008-2652 19567536

[29] Delange F. Optimal iodine nutrition during pregnancy, lactation and the neonatal period. Int J Endocrinol Metab. 2004; 2:1–12.

[30] Bouhouch RR, Bouhouch S, Cherkaoui M, Aboussad A, Stinca S, Haldimann M, et al Direct iodine supplementation of infants versus supplementation of their breastfeeding mothers: a double-blind, randomised, placebo-controlled trial. Lancet Diabetes Endocrinol. 2014; 2(3):197–209. 10.1016/S2213-8587(13)70155-4 24622750

[31] Zimmermann MB. The adverse effects of mild-to-moderate iodine deficiency during pregnancy and childhood: a review. Thyroid. 2007; 17(9):829–835. 10.1089/thy.2007.0108 17956157

[32] Antonangeli L, Maccherini D, Cavaliere R, Di Giulio C, Reinhardt B, Pinchera A, et al Comparison of two different doses of iodide in the prevention of gestational goiter in marginal iodine deficiency: a longitudinal study. Eur J Endocrinol. 2002; 147(1):29–34. 10.1530/eje.0.1470029 12088916

[33] Pedersen KM, Laurberg P, Iversen E, Knudsen PR, Gregersen HE, Rasmussen OS, et al Amelioration of some pregnancy-associated variations in thyroid function by iodine supplementation. J Clin Endocrinol Metab. 1993; 77(4):1078–1083. 10.1210/jcem.77.4.8408456 8408456

[34] Rohner F, Zimmermann MB, Jooste P, Pandav C, Caldwell K, Raghavan R, et al Biomarkers of nutrition for development—iodine review. J Nutr. 2014; 144(8):1322S–1342S. 10.3945/jn.113.181974 24966410 PMC4093988

[35] Zimmermann MB, Aeberli I, Andersson M, Assey V, Yorg JA, Jooste P, et al Thyroglobulin is a sensitive measure of both deficient and excess iodine intakes in children and indicates no adverse effects on thyroid function in the UIC range of 100–299 mug/L: a UNICEF/ICCIDD study group report. J Clin Endocrinol Metab. 2013; 98(3):1271–1280. 10.1210/jc.2012-3952 23345097

[36] Ghirri P, Lunardi S, Boldrini A: Iodine supplementation in the newborn. Nutrients 2014; 6(1):382–390. 10.3390/nu6010382 24448111 PMC3916868

[37] Zimmermann MB, Adou P, Torresani T, Zeder C, Hurrell R: Persistence of goiter despite oral iodine supplementation in goitrous children with iron deficiency anemia in Cote d'Ivoire. Am J Clin Nutr. 2000; 71(1):88–93. 10.1093/ajcn/71.1.88 10617951

[38] Lazarus JH, Parkes AB, John R, N'Diaye M, Prysor-Jones SG. Endemic goitre in Senegal—thyroid function etiological factors and treatment with oral iodized oil. Acta Endocrinol. 1992; 126(2):149–154. 10.1530/acta.0.1260149 1543020

[39] de Escobar GM, Obregon MJ, del Rey FE. Iodine deficiency and brain development in the first half of pregnancy. Public Health Nutr. 2007; 10(12A):1554–1570. 10.1017/S1368980007360928 18053280

[40] Melse-Boonstra A, Gowachirapant S, Jaiswal N, Winichagoon P, Srinivasan K, Zimmermann MB. Iodine supplementation in pregnancy and its effect on child cognition. J Trace Elem Med Biol. 2012; 26(2–3):134–136. 10.1016/j.jtemb.2012.03.005 22575544

[41] Vermiglio F, Lo Presti VP, Moleti M, Sidoti M, Tortorella G, Scaffidi G, et al Attention deficit and hyperactivity disorders in the offspring of mothers exposed to mild-moderate iodine deficiency: a possible novel iodine deficiency disorder in developed countries. J Clin Endocrinol Metab. 2004; 89(12):6054–6060. 10.1210/jc.2004-0571 15579758

[42] Rose SA, Feldman FJ, Jankowski JJ. Infant visual recognition memory. Dev Rev. 2003; 24:74–100. 10.1037/0012-1649.39.3.563 12760523

[43] Colombo J, Wayne DM. Infant visual habituation. Neurobiol Learn Mem. 2009, 92:225–234. 10.1016/j.nlm.2008.06.002 18620070 PMC2758574

[44] Oakes LM, Kovack-Lesh KA. Infants' visual recognition memory for a series of categorically related items. J Cogn Dev. 2013; 4(1):63–86. 10.1080/15248372.2011.645971 23495291 PMC3593603

[45] Colombo J, Kannass NK, Shady JD, Kundurthi S, Maikranz MJ, Anderson JC, et al Maternal DHA and the development of attention in infancy and toddlerhood. Child Dev. 2004; 75:1254–1267. 10.1111/j.1467-8624.2004.00737.x 15260876

[46] Jacobson SW, Jacobson JL, Sokol RJ, Martier SS, Ager JW. Prenatal alcohol exposure and infant information processing ability. Child Dev. 1993; 64(6):1706–1721. 8112114

[47] Jacobson SW, Jacobson JL, Sokol RJ, Martier SS, Chiodo LM. New evidence for neurobehavioral effects of in utero cocaine exposure. J Pediatr. 1996; 129:581–590. 10.1016/s0022-3476(96)70124-5 8859266

[48] Bautista A, Baker AP, Dunn TJ, Sanchez M, Kaiser LD. The effects of oral iodized oil on intellignece, thyroid status, and somatic growth in shcool-age children from an area of endemic goiter. Am J Clin Nutr. 1982; 35:127–134. 10.1093/ajcn/35.1.127 6278919

[49] Murcia M, Rebagliato M, Iniguez C, Lopez-Espinosa MJ, Estarlich M, Plaza B, et al Effect of iodine supplementation during pregnancy on infant neurodevelopment at 1 year of age. Am J Epidemiol. 2011; 173(7):804–812. 10.1093/aje/kwq424 21385833

[50] Perkins JM, Kim R, Krishna A, McGovern M, Aguayo VM, Subramanian SV: Understanding the association between stunting and child development in low- and middle-income countries: Next steps for research and intervention. Soc Sci Med. 2017; 193:101–109. 10.1016/j.socscimed.2017.09.039 29028557

[51] Helmizar H, Jalal F, Lipoeto NI, Achadi EL: Local food supplementation and psychosocial stimulation improve linear growth and cognitive development among Indonesian infants aged 6 to 9 months. Asia Pac J Clin Nutr. 2017; 26(1):97–103. 10.6133/apjcn.102015.10 28049268

[52] Zimmermann MB, KÖhrele J. The impact of iron and selenium deficiencies on iodine and thyroid metabolism: biochemistry and relevance to public health. Thyroid. 2002; 12 (10):867–78. 10.1089/105072502761016494 12487769

