# Supplementary material for: Iodine supplementation of lactating women and assessment of infant visual information processing and maternal and infant thyroid function: A randomized trial
Source: PLoS One. 2019 Oct 7;14(10):e0223348. doi: 10.1371/journal.pone.0223348 (PMC6779247; doi:10.1371/journal.pone.0223348)
Supplement: S1 File — (PDF) [file pone.0223348.s001.pdf]

S1 File. HANDWRITTEN FORMS WILL NOT BE ACCEPTED

APPLICATION MUST BE SINGLE SIDED

APPLICATION FOR REVIEW OF HUMAN SUBJECTS RESEARCH

SUBMITTED TO THE
OKLAHOMA STATE UNIVERSITY INSTITUTIONAL REVIEW BOARD
Pursuant to 45 CFR 46

____
IRB Number

FOR OFFICE USE ONLY

Title of Project: Community iodized salt distribution & Visual Information Processing (VIP) of Infants at 6 months of age

Is the Project externally funded? ☐Yes ☒No If yes, complete the following: ☐Private ☐State ☐Federal

Agency: Nestle Foundation Grant No: OSU Routing No:

Type of Review Requested: ☐ Exempt ☐ Expedited ☒ Full Board

Principal Investigator(s): *I acknowledge that this represents an accurate and complete description of my research. If there are additional PIs, provide information on a separate sheet.*

Tafere G/Egziabher Belay		11/21/11
**Name of Primary PI** (typed)	Signature of PI	Date
Nutritional sciences	Human Sciences	
Department	Col ege	
301 HS	4057623112	taferege@yahoo.com
PI's Address (Street, City, State, Zip)	Ph ne	E-Mail

Required IRB Training Complete: ☒ Yes ☐ No
(Training must be completed before application can be reviewed)

**Name of Co-PI** (typed)	Signature of Co-PI	Date
Department	College	
I's Address	Phone	E-Mail

Required IRB Training Complete: ☐ Yes ☐ No
(Training must be completed before application can be reviewed)

Adviser (complete if PI is a student): *I agree to provide the proper surveillance of this project to ensure that the rights and welfare of the human subjects are properly protected.*

Dr. Barbara J. Stoecker		11/21/11
**Adviser's Name (typed)**	Signature of Adviser	Date
Nutritional Sciences	Human Sciences	
Department	College	
301 HS	744-5038	Barbara.stoecker@okstate.edu
Adviser's Address	Phone	E-Mail

Required IRB Training Complete: ☒ Yes ☐ No
(Training must be completed before application can be reviewed)

NOTE: If sufficient space is not provided below for a complete answer in sufficient detail for the reviewer to fully understand what is being proposed, please use additional pages as necessary.

1. Describe the purpose and the research problem in the proposed study. *Your response in this section will enable the reviewers to determine whether the project meets the criteria of research with human participants and also the extent to which the research may produce new generalizable knowledge that may benefit the participants and/or society.*

In Ethiopia, iodine deficiency disorder (IDD) is a serious public health problem in all age groups. Iodine deficiency can cause serious consequences on brain and physical development called endemic cretinism which is characterized by mental retardation, severe and irreversible alterations in brain development, and multiple neurological signs including deaf mutism, motor rigidity, and shuffling gait. Infants under 2 years of age are one of the most vulnerable groups to be affected by IDD other than pregnant and lactating mothers. In neonates iodine deficiency disorders (IDD) could cause neonatal goiter, neonatal hypothyroidism, or endemic mental retardation. Maternal thyroid dysfunction could lead to developmental impairment in the offspring. A mild to moderate iodine deficiency in pregnancy may affect children's cognitive and motor function. Therefore special attention has been given to pregnant and lactating women and neonates regarding preventing and controlling iodine deficiency because of the impact of maternal, fetal and neonatal hypothyroxinemia on brain development of the offspring.

However, the impact of iodine supplementation to lactating mothers on the infant's iodine status and visual information processing (VIP) is not known. Awareness of iodine deficiency is very low in the rural areas of southern Ethiopia and those who are aware that iodine is important associate its deficiency with goiter but not with an effect on a child's ability to learn. If beneficial effects of iodine on VIP are demonstrated, the results will be beneficial in educating mothers about the importance of iodized salt and will put pressure on the government to speed their salt iodization programs.

The purposes of this study are 1) to conduct an experimental study on the effect of iodine supplementation to lactating mothers on visual information processing (VIP) of their 6 month old infants. VIP is a type of visual recognition memory which is based on measuring novelty preference, processing speed, longest look and shift rate of infant's vision against familiar versus novel stimulus. Infant's visual recognition memory is the beginning of cognitive effort which can predict broad cognitive abilities in later childhood. and 2) to evaluate the effectiveness of Ethiopia's new salt iodization program in delivering adequately iodized salt at the village level and to assess impacts of availability of iodized salt and community iodine awareness on urinary iodine concentration (UIC) of a random sample of community members.

2. (a) Describe the subjects of this study:

1) Describe the sampling population: For **Objective 1**, the sampling population will be limited to lactating mothers (18 years and older) and their infants. Participants for this study will be selected from all mothers with 6-month-old infants at baseline (*control* groups) and from all mothers of newborn infants (*treatment* groups). The mother-infant dyads will be recruited from two local communities (kebeles) as described in sections 2 & 3.
For **Objective 2**, families (households) will be selected from a third community. The sampling population for both objectives will be from Sidama Zone in southern Ethiopia. The study population depends on subsistence farming for their livelihood. Their major staple food is enset (*Enset ventricosum*) followed by unrefined maize. The area is known for multiple

micro-nutrient deficiencies and iodine deficiency is one of the major prevalent problems.

2) Describe the subject selection methodology (i.e. random, snowball, etc.): For **Objective 1**, all women in the selected communities with infants of the requisite ages (See Questions 3 and 4) will be invited to participate. Recruitment will continue at ~two week intervals until the sample is complete. For **Objective 2**, families will be randomly selected from the lists of households obtained in the kebele (community) office. Additional families will be drawn from this list as needed to complete the sample size of 40 households.
3) Describe the procedures to be used to recruit subjects. Include copies of scripts, flyers, advertisements, posters or letters to be used. **If recruitment procedures will require access to OSU System email addresses you will need to include Appendix A of this application**:

As part of their rural health system, Ethiopia identifies community health workers (CHWs) who live in each rural community. These CHWs are expected to have a census of persons living in the village and to advise community members of upcoming events related to health and well-being such as immunizations days, latrine building projects, etc. We will work through these CHWs which is the method approved by the regional government.

Copies of scripts to be used by community health workers (CHWs) and the research team to recruit participants are attached. No fliers, advertisements, posters or letters will be used to avoid sample bias because many of the community adults have not had opportunities for formal education; thus written announcements would bias the sample towards the more economically and socially advantaged.

After obtaining Institutional Review Board approvals from Hawassa University and permission from the regional government, the next step in introducing a new project to the community is a thorough discussion of the projects aims and needs with the CHWs and any other interested community officials. CHWs will be given a copy of the scripts and consent forms for their community. After the CHWs thoroughly understand the project, they will be asked to spread the word about community meeting(s) to all mothers of 6±0.25 month old infants. The researcher (TG) will be present at all of the community meetings. The script will be read at each meeting and all questions will be answered. Our standard procedure is to allow volunteers to consent after that meeting or to go home and return on another scheduled day to volunteer (or ask more questions). We ask that CHWs use the script to provide women with details about the upcoming meeting and project, but we are aware that some information about the project spreads through the village by word of mouth. This is the reason that we will ask all interested mothers of 6 month old infants to attend a group meeting for recruitment. These group meetings will continue at approximately two week intervals until the required number of mothers of 6 month old infants are recruited for the study. These infants will comprise the *control* groups.

Recruitment of the *treatment* groups (infants age 0-1 week) will also be through the CHWs who will be aware of infant births in the community and will approach mothers about participation. They will initially tell women who are within a week of delivery about the study using the script. However, we will not expect women to walk to a community meeting within one week of delivery. Therefore the researcher will accompany the CHW to homes of all women interested in participating in the study. The study will be explained using the script, and consent will be obtained if

the mother wishes to participate. We are aware that information about the study will also spread by word of mouth throughout the village, but the researcher will be certain that women recruited have understood the script and he will personally obtain their consent in the presence of a community member. Study enrollment will continue until the required sample size has been attained.

Recruitment for **Objective 2** will also be with the assistance of the CHWs. A randomized sample of families will be drawn from the list maintained by the CHWs in the kebele office and the CHWs will visit each of these households and explain the study using the script. The heads of these households (and other interested family members) will be invited to attend community information meetings if they are interested in participation. The researcher will attend the information meetings and ensure that the script is read and that all questions are answered. He will obtain consent at that meeting or will invite the household head to return at a later time to give consent if preferred. All members of the household will be invited to participate in the study. Parental consent will be required for participation of anyone under 18 years of age. Children 12-18 years of age will also provide assent for participation. (Cultural norms give Ethiopian children less freedom to disagree with parental decisions so children under 12 will be accepted into the study on the basis of their parent's consent, however, we will not force any child to participate in the study (provide a urine sample) against their will. Additional families will be drawn from the community list and invited to participate to replace any families that do not choose to participate (to reach the sample size of 40 households). If 50% of the household members participate, that family will be included in the study.

4) How many subjects are expected to participate?: ***For Objective 1,*** a sample size of 160 subjects was calculated using an alpha of 0.05, 80% power and an effect size of 0.25. From these, 80 mothers and their 6 month old infants from two kebeles will be tested to serve as *controls* (40 from each kebele). Immediately after testing, their mothers will receive iodized salt in one village or an iodized oil supplement in the other village. *Treatment groups*: Subsequently 40 women will be identified for iodized oil supplements within one week of delivery in the "iodized oil" kebele and 40 will be identified in the "iodized salt" kebele. The mothers in the iodized salt kebele will continue to receive iodized salt for themselves and their family for six months. Infants in both kebeles will have VIP testing at 6 months of age. For **Objective 2**, 40 families (approximately 280 people) will provide urine samples at specific intervals.

What is the expected duration of participation for each segment of the sampling population? If there is more than one session, please specify the duration of each session: **Objective 1**: For the two *control* groups in addition to consent, the mothers will be involved in answering a questionnaire, blood, breast milk and urine sample collection, anthropometry and a goiter check and cognitive tests, urine and blood collection of their infants. These will take approximately two hours. For the two *treatment* groups the mothers will be tested at baseline, after 3 months and at 6 months. At baseline, mothers will be involved in blood, milk and urine sample collection, anthropometry and a goiter check in addition to consent. These will take approximately one hour. At 3 months, urine, breast milk and blood of the mothers and urine of the infants will be collected. These will take approximately forty minutes. At 6 months, the mothers will be involved in answering a questionnaire, blood, breast milk and urine sample collection, cognitive tests, urine and blood collection of their infants, anthropometry and a goiter check. These will take approximately one hour. **For Objective 2.** The household head will answer a questionnaire at baseline and

identify all household members. This visit will take approximately 1 hour. Urine will be collected from all consenting members of the household at baseline, 1 and 6 months. Time required per individual will be about 5-10 minutes.

5) Describe the calendar time frame for gathering the data using human subjects: September 2012 to May 2013

6) Describe any follow-up procedures planned: Nutrition education sessions will be provided in each community related to use of iodized salt and consequences of iodine deficiency. We will also teach the women's associations to monitor the quality of iodized salt at the household level using rapid test kits obtained through UNICEF for local monitoring operations.
These follow-up procedures are not an integral part of this research per se, but they are an integral part of procedural protocols for Hawassa University's Nutrition Research Unit, so we are committed to following up with education for the community.

(b) Are any of the subjects under 18 years of age? ☒ Yes ☐ No

If Yes, you must comply with special regulations for using children as subjects. Please refer to IRB Guide.

3. Provide a detailed description of any methods, procedures, interventions, or manipulations of human subjects or their environments and/or a detailed description of any existing datasets to be accessed for information. Please indicate the physical location where the research will take place (if applicable). Include copies of any questionnaires, tests, or other written instruments, instructions, scripts, etc., to be used.

Please see **Tables 1-3** which have been attached to this application and which may provide more clarity about the procedures for this project.

This research will be conducted in rural communities in Sidama zone of southern Ethiopia. Work done by Hawassa University's Nutrition Research Unit has demonstrated the presence of serious iodine deficiency but surveys found that <5% of the village population was aware of the importance of iodine for health. The Federal Government of Ethiopia is beginning to promote the use of iodized salt, but this information is not yet reaching rural residents in the Sidama zone.

To conduct this research, the team will be led by Tafere G/Egziabher, a faculty member at Hawassa University, who is currently conducting research toward his Ph.D at OSU. After obtaining approvals from the Institutional Review Board at Hawassa University and from the regional government for the Sidama zone, he will begin arrangements for the study in the rural communities. In the community, he will coordinate with the CHWs, who in the Ethiopian rural health system are the persons charged with providing basic health and sanitation information to the population. These CHWs come from the community, and are expected to know all families in their community and to know which women are pregnant and which have recently delivered. Tafere will be accompanied to the village by the medical technologist if blood samples are to be drawn. When Tafere is doing VIP testing he will be accompanied by two assistants or data collectors. One of these will administer the questionnaire to the mothers and the other will assist with setting up and managing the pictures being shown to the infant and coding and storing the urine and milk samples. The CHWs will also assist with co-ordinating appointments as needed.

The Nutrition Research group at Hawassa University has a great deal of experience in handling studies involving human data including more than five years of NIH-funded research. They are thus quite well versed in confidentiality issues. However, re-training is provided on all aspects of any new project. As part of the confidentiality training, they will be told that all of the data collected from all participants are personal and confidential that they

cannot talk about the information to another person or to each other. To keep the information confidential, questionnaires will be collected by the PI immediately after information is gathered at the site. These questionnaires will be returned to locked file cabinets in the Nutrition Research Unit each evening. Participant names will be removed from the upper right hand corner of the questionnaires as soon as the names and code numbers are cross checked.

For **Objective 1**, two rural communities will be selected. In one of these kebeles, *treatment* will be iodized oil supplementation of the mother and in the other the *treatment* will be provision of iodized salt. Participants for this study will be selected from all mothers with 6-month-old infants at baseline and from all mothers of newborn infants. All of the mothers that will be included in the study will be 18 years and older.

At baseline, *control* groups will be established by recruiting mothers of forty 6-month-old infants in each of the two kebeles. Mother-child dyads will be registered and infants' visual information processing (VIP) will be tested. Then these *control* mothers will be given iodized salt for themselves and their family for six months. For the *treatment* groups, 80 women (40 from each kebele) will be recruited for the study within one week of delivery. In one kebele mothers will be supplemented with 250 µg/day of iodine as an iodized oil capsule for 6 months (per the recommendations of the World Health Organization for treatment in iodine deficient areas) beginning within one week of delivery. In the other kebele, 40 women will be provided with iodized salt for themselves and their family within one week of delivery. The women in the kebele receiving iodized salt will continue to receive iodized salt until their infant is 6 months old. After 6 months, VIP will be tested on the 80 infants whose mothers received supplemental iodine as iodized oil or as iodized salt. These results will be compared with their respective *control*s collected at baseline.

Objective 2 will evaluate the efficacy of testing iodized salt by a women's association in a third kebele. At baseline, urinary iodine concentration (UIC) will be measured on a random sample of all consenting members from 40 families. Iodized salt for all families will be distributed free for one month and participants will be encouraged to continue purchase of iodized salt in the market. UIC will be tested at baseline and re-tested at 1 and 6 months.

For Objective 1:

<u>Data collection</u>

In each community data collection will take place at the community health post or at the home of the participants as appropriate to the context of the study. Tafere G/Egziabher will go to the communities at least twice a week to collect data as the infants reach the appropriate ages. He is fluent in both English and Amharic. He will be accompanied by at least one of the trained researchers from Hawassa University's Nutrition Research Unit and by a medical technologist who is also fluent in English and Amharic. For the *control* groups, mother-infant dyads will come to the community health post when the infant is 6 months old. For the *treatment* groups, samples will be collected from mother-infant dyads at home at baseline and the mother will be asked to bring her infant to the community health post at 3 and 6 months at an appointed time. Data will be collected according to the following procedures.

<u>Maternal history and demographic data</u>

Data to be collected as part of a general history including community of residence; name; age (best estimate); breastfeeding status; child deaths; family size, age at marriage, number of live births, antenatal care, prenatal supplements and mother's assessment of child's health. This questionnaire will be administered to individually to mothers when their infant is six months of age by a member of the research team. The questionnaires contain a place for mother's name in the upper right hand corner because it is culturally appropriate to address the participants by name rather than number. However, names will be cut off of the questionnaires, leaving only the code number, as soon as the data are checked for

completeness at the end of the day.

Socioeconomic Status (SES)

SES will be assessed by a combination of parental education and family wealth. Wealth criteria will include maternal possession of bicycle, lantern, or mobile phone and family ownership of animals, crops and land size; family size, type of home & windows, ownership of umbrella and shoes; children attending school; outside employment, water source and latrine.

Visual information processing (VIP). For VIP measures, eight pictures of young adult Ethiopian faces without emotional expression (four male and four female) will be used. Two laptop computers and a video projector will be used to project the pictures on a blank screen. One computer will control the presentation of stimuli. A second computer that is attached to a video camera will be used to follow and record the infant's looking behavior. Although the infant's looking behavior will be coded live, the infant's looking process will also be recorded on this second computer for later reliability testing.

A quiet room will be prepared for the VIP data collection. The infant will sit on mother's lap in front of the camera, eyes forward, and half a meter away from the screen. The researcher will sit behind the blank screen but fully covered by and out of the sight of the infant. The researcher will adjust the visibility and focus of the infant's face and eyes on the laptop that is connected to the camera. Once the focus is adjusted, the familiarization phase will begin. A randomly-selected picture will be shown on the screen in front of the infant. The researcher will follow the infant's look on the laptop and code these looks. If the infant is fussy, their data would not be useful and they will not be forced to continue the study. At the mother's request the child could be scheduled for the VIP later in the day or the next day that the researcher will be in the area.

Anthropometry measurement

As a measure of women's nutritional status, weight and height will be measured to calculate body mass index (BMI = Wt (Kg)/Ht^2 (m) and mid upper arm circumference (MUAC) will be measured. Each woman's weight will be measured on a solar digital scale (Uniscale, UNICEF, NY) and recorded to the nearest 100 grams and height will be measured to the nearest 0.1 cm using a single calibrated instrument (Adult Board, Schorr Productions, Olney, MD).

As a measure of infant's nutritional status, head and upper arm circumference, weight and length will be measured. Infant's weight-for-age, length-for-age, weight-for-length and head and upper arm circumference will be converted to Z-scores using WHO Anthro software.

Biomarkers

All biological samples will be coded by number only. They will be collected on ice in the field and taken to Hawassa University's Nutrition Research Unit for storage in a -20°C freezer. At the conclusion of data collection, biological samples will be shipped on dry ice by FedEx or DHL to the Nutritional Sciences laboratories at Oklahoma State University for analysis. The appropriate shipping permits will be obtained through the Centers for Disease Control.

Mothers will collect urine in a cup and urine samples will be obtained from infants by squeezing urine out of cotton balls in a disposable diaper. Urine samples will be transferred to fill tightly sealed vials and frozen for subsequent assessment of urinary iodine concentration. From a heel prick of infants, two drops of blood will be collected on filter paper for TSH and retinol analysis and a drop of blood will be collected on a slide for hemoglobin analysis. Ten ml of breast-milk will be collected from each mother for assessment of iodine concentration. A fasting morning venipuncture blood sample (10 ml) will be collected from each participant by an experienced medical technologist. Plasma will be used for analysis of thyroglobulin

(Tg), thyroid hormone concentrations (T_3 and T_4) and thyroid stimulating hormone (TSH).

Goiter

Goiter of each women will be determined by a health professional based on the following grades: grade 0, no palpable or visible goiter; grade 1, goiter palpable but not visible when neck is in the normal position; grade 2, goiter visible when neck is in the normal position

Diet, supplementary feeding and health

- Diet based questions will be asked in order to evaluate food diversity and to identify food substances containing goitrogenic compounds. Health indicators, food consumption pattern and supplementary feeding of their infants will also be assessed.

Iodized salt

Salt will be purchased from the local market and women's associations will be taught to test salt samples for iodine content with rapid test kits (UNICEF). Iodized salt will be distributed for 6 months without cost to the 80 mother-infant dyads who participate with their infants in the VIP testing in the iodized salt kebele and to the 40 mother-infant dyads who participate with their infants in the control groups in the iodized oil kebele (The 40 mothers in the iodized oil kebele will receive a daily dose of iodized oil for six months). Teaching the women's associations to test salt for iodine will create awareness in the community and will ensure that community dwellers are actually purchasing iodized salt in the market.

Iodized oil

Iodized oil capsules that contain 250 µg iodine will be purchased through UNICEF and stored in a refrigerator at Hawassa University until all are administered individually to the 40 mothers in the iodized oil kebele by the PI. The quantity of iodine is based on recommendations from the World Health Organization and has been used in iodine deficient areas around the world.

For Objective 2:

The research team will go to the home of participating households at an appointed time and will take with them the supplies for collection of urine samples from all consenting family members. A questionnaire will be administered to the household head by TG or another researcher fluent in Amharic.

Socioeconomic Status (SES) and salt utilization

SES will be assessed by a combination of parental education and family wealth. Wealth criteria will include maternal possession of bicycle, lantern, or mobile phone and family ownership of animals and crops and land size; family size, type of home & windows, ownership of umbrella and shoes; children attending school; outside employment, water source and latrine. Questions about purchase and handling of salt will also be included.

Urinary Iodine

Adults and children will be asked to collect a urine sample in a cup at baseline, 1 month and 6 months. Urine will be collected from infants and very young children by squeezing urine out of cotton balls in a disposable diaper. Urine samples will be transferred to fill tightly sealed vials identified only by number and frozen for subsequent shipment to OSU for assessment of urinary iodine concentration.

Data storage

All data will be stored at Hawassa University (HU) until transported to Oklahoma State

University for analysis. The Nutrition Research Unit at HU has been doing NIH-funded research for more than five years as well as monitoring research projects for various international organizations. Names will be removed from questionnaires and the questionnaires will be stored in locked filing cabinets in an office in the Nutrition Research Unit. Faculty and graduate students have been thoroughly trained in confidentiality issues but confidential handling of data is always reviewed before a new project begins. Only numbers will appear on all biological samples. No names will be entered into databases.

Role of the Women's Associations

Working with the women's associations will raise awareness of the importance of iodine and will give the community some assurance that they are actually purchasing iodized salt. The iodized salt test kits are designed for use in this type of setting. A small measure of salt is dissolved in water and a strip is inserted in the solution. The degree to which the strip turns purple is compared to a color scale which gives an estimate of the quantity of iodine in the salt. Among the leaders of the women's association, there will be women who have had sufficient formal education to easily handle this salt testing in the village.

Research Team

Tafere G/Egziabher will lead the research team. The number of assistants he will need from the Nutrition Research Unit will depend on the schedule for the day. Assistants employed through the Nutrition Research Unit usually have at least high school education and many have a two-year diploma. It is unlikely that he will hire anyone who has not previously participated in on-going students done by the Nutrition Research Unit. On days that he does testing of infants for visual information processing he is likely to need two assistants from Hawassa University as well as help from a CHW. One of these assistants will administer questionnaires. One will coordinate storage of the biological samples. If there is blood to be drawn, he will be accompanied by the medical technologist. The medical technologist who usually accompanies the team is well known and respected in the villages based on his many years of service at a local clinic. In addition to drawing blood he will document visible goiter or palpate the thyroid gland to evaluate the presence of less obvious goiter. His training as a health professional has prepared him for these roles. The CHW will organize participants as they appear for appointments. Tafere will provide overall management of the team, assist with the transfer of samples to storage containers, and monitor the data for quality control.

Scheduling of appointments

Appointments will be scheduled through the CHW. There is a weekly market day and the appointment might be made for before or after that day. The participant will be asked to come at a general time such as early morning, mid-morning or at a specific time if they have a watch.

Sample collection

In terms of privacy, the women will be given cups and allowed to go to the latrine near the health center to collect the urine sample. Women will also be given cups for collection of a breast milk sample and can go to a more private location behind the community health post to collect the breast milk sample. They know how to express breast milk, so it is not something that we have to teach them. Women nurse their

babies openly and do not have the cultural reservations about breast feeding that are sometimes seen in the west.

Advice related to goiter

If a person has a goiter they will be told. Sometimes persons with palpable but not visible goiter do not realize that they have a goiter. They will be told that goiter is not caused by drinking dirty water (a common belief) and that goiter is more than just a cosmetic problem. They will be encouraged to consume iodized salt as a way to prevent goiter. Persons with large goiter will be encouraged to consult with the local health clinic for followup.

4. Will the subjects encounter the possibility of stress or psychological, social, physical, or legal risks that are greater than those ordinarily encountered in daily life or during the performance of routine physical or psychological examinations or tests? ☐Yes ☒No

The reason that salt is used worldwide as a vehicle for delivery of iodine is that people do not consume vastly different amounts of salt and thus the dose of iodine obtained from salt is fairly consistent. The Ethiopian government has mandated that all salt sold in the market should be iodized and universal salt iodization (USI) was implemented effective February 2012. USI is the iodization of all salt in the country at the production site. However due to the fact that iodine content of salt can decrease from improper handling, storage and utilization, monitoring the iodized salt actually reaching the household members is essential. Awareness of signs of IDD and its effects on cognition of children may be particularly viable ways to get the attention of parents and policy makers. We hope this project and our data will contribute to consumer and governmental awareness of the continuing importance of addressing Ethiopia's iodine deficiency problem.

If Yes, please justify your position:

5. Will medical clearance be necessary for subjects to participate because of tissue or blood sampling, administration of substances such as food or drugs, or physical exercise conditioning? ☒Yes ☐N

If Yes, please explain how the clearance will be obtained: The medical technologist who draws the blood sample will not take a sample from a mother who has a fever or other clinical signs of illness. Also if her infant has fever, rasping cough, or severe (>3/day) diarrhea, the mother-child dyad will not be enrolled in the study or provide samples on that day.

6. Will the subjects be deceived or misled in any way? ☐Yes ☒No

If Yes, please explain:

7. Will information be requested that subjects might consider to be personal or sensitive? ☒ Yes ☐No

If Yes, please explain: The socio economic status (SES) data and anthropometrics might be considered personal; however, the information is important for the study and training will be given to the research team (data collectors) by the PI at Hawassa University. Furthermore, the nutrition group at Hawassa University has a great deal of experience in handling studies involving human data and are quite well versed in

confidentiality issues. As part of the training they will be told that all of the data collected from all participants are personal and confidential that they cannot talk about the information to another person or to each other. To keep the information confidential, questionnaires will be collected by the PI immediately after information is gathered at the site. These questionnaires will be returned to locked file cabinets in the Nutrition Research Unit each evening. For urine and milk collections, the women will be given cups and sent to the latrine or a private place near the health center to collect the sample. Collecting these types of samples does not seem to be in any way offensive to this population group.

8. Will the subjects be presented with materials that might be considered to be offensive, threatening, or degrading? ☐Yes ☒No

If Yes, please explain, including measures planned for intervention if problems occur.

9. Will any inducements be offered to the subjects for their participation? ☒Yes ☐No

If Yes, please explain: Small gifts such as a picture, hair oil or a head scarf will be given to the mothers and t-shirts will be given for the infants. Moreover, iodized salt will be given for their household consumption.

The price of iodized salt is controlled by the government and is not more expensive than non-iodized salt, so providing iodized salt is not an excessive inducement. We are confident that participants who receive the iodized salt in our study would not be at risk of being robbed or extorted, because it is not an item that as substantial monetary value. The ethical issues associated with working with families who are iodine deficient and not providing iodized salt far outweigh any risk of excessive inducement in our opinion.

NOTE: If extra course credit is offered, describe the alternative means for obtaining additional credit available to those students who do not wish to participate in the research project.

10. Describe the process to be used to obtain the consent/assent of all subjects including (as appropriate); who will seek the consent/assent, steps to minimize coercion or undue influence, and the method(s) to be used to document the consent.

Please provide copies of all consent documents with your application

A written consent form (copies attached) will be used, but because most of the participants are illiterate the consent will be read in their local language and they will have the option of signing by finger print. As discussed in the subject recruitment section, the CHWs will make the initial contacts with potential participants. These potential participants will be invited to participate in an information meeting. They can choose to come or not to come to the information meeting and the recruitment script makes it clear that they do not have to participate. If they come to the information meeting, they can be read the consent form and sign it after that meeting but they are also completely free to come back on another day to sign the consent or not to participate at all. One reason that research projects conducted through Hawassa University suggest that potential participants are free to return on another day (after the information meeting) to give consent is so that participants will not feel pressured to participate in the study.

We have allowed 9 months for data collection for this study which based on birth rate in the communities is more than ample time to involve many more participants than we need. Data from a fussy infant will not be useful to us, so we certainly have no reason to force an infant to participate.

For consent/assent for the study comprising Objective 2, a family will also

have no reason to coerce family members to participate. Because all that the family members are being asked to do is provide urine samples, it is unlikely that they will find this to be particularly onerous.

For Objective 1. The consent form will include the mother and her infant and the mother will sign for her infant as well because the infant is too young to provide assent. If the infant is too fussy to look at the computer screen, they will be excluded from the study. If the mother wishes, the infant be rescheduled later in the day or the next day, but we are not anxious to try to collect data from a fussy infant nor are we going to coerce anyone to participate. We do not plan to reschedule a mother-infant dyad more than one time. Hawassa University's Nutrition Research Unit has been working in these villages for many years and they have an excellent reputation with the villagers based on the quality of the information they provide as well as the way they interact with research participants. Tafere G/Egziabher is a faculty member at Hawassa University and he has no desire to sacrifice that carefully maintained relationship.

For Objective 2: The household head will sign a consent form and give the names of all household members approved for participation in the urine collection. The household head will give permission for participation for all children on the list who are under 12 years of age, but we will not force any child to participate. Adults from this list will sign individual consent forms. Children on the list between the ages of 12-18 will sign individual assent forms. These forms are attached to this application.

All scripts and consent forms are translated into Amharic. These Amharic forms will be used. The CHW does not need to be fluent in English but instead will be utilizing the translated forms.

11. Are you requesting a waiver of documentation of consent (no signature on consent/assent forms)? If you are conducting an anonymous survey, online or in paper form, check yes here.

☐Yes ☒No

If yes, provide a justification for waiving documentation based on one of the two criteria allowing the waiver.

12. Do you wish to waive of some of the elements of consent/assent or parental permission or the entire consent/assent or parent permission process?

☐Yes ☒No

If yes, provide a justification for the waiver that addresses each of the criteria that must be met for the waiver to be allowed.

13. Will the data be a part of a record that can be identified with the subject? ☒Yes ☐No

If Yes, please explain: Participants will be assigned a code number. Tafere G/Egziabher will maintain this list locked in his office. The name/number list will be used for crosschecking data and for locating participants in their homes for the followup urine, blood and breastmilk samples. Names will be cut off any questionnaires as soon as data

are checked. No names will be entered into the data base and it will be password protected. The list with names and identifiers of the study subjects will be kept in a locked file in the Nutrition Research Unit at Hawassa University. The identifier list will be destroyed as soon as the data have been checked and cleaned (at least by Sep. 1, 2013). All will be destroyed after publication of data or no later than three years after the end of the study. Biological samples will be incinerated as soon as analyzes are completed and checked.

14. Describe the steps you are taking to protect the confidentiality of the subjects and how you are going to advise subjects of these protections in the consent process. Include information on data storage and access. If data will not be reported in the form of group means, please explain how the data will be reported.

The questionnaires and VIP tests will be administered to the participants individually and portable screens or separate rooms will be used as necessary to increase privacy. Only the investigators will have access to the data. Data will be password protected. The participants will be assured that their individual information will not be shared with anyone outside of the research team and data will be reported as a group. The Nutrition Research Unit at Hawassa University has locked files. Questionnaries, consents and assents will be brought to these files every evening as the research team return from the village and processes the biomarkers. Data will be stored until publication of papers is complete. (no longer than three years).

15. Will the subject's participation in a specific experiment or study be made a part of any record available to his or her supervisor, teacher, or employer? ☐Yes ☒No

If Yes, please describe:

16. Describe the benefits that might accrue to either the subjects or society. *Note that 45 CFR 46, Section 46.111(a)(2) requires that the risks to subjects be reasonable in relation to the anticipated benefits. The investigator should specifically state the importance of the knowledge that reasonably may be expected to result from this research.*

The outcome of this study will help assess iodine status of the women and their infants as well as the relation to cognition performance of the infants. Based on the result, nutrition education will be provided to the participants and to health extension workers working with the study population as well as to other communities that have similar food habits. Moreover, iodized salt will be given to the subjects and their families. Information gained about mean iodine status will also be shared with other appropriate stakeholders. We believe that the only way to force action on the government's part to provide enough iodized salt for its population is to build consumer awareness, and we hope that our data and our nutrition education efforts will contribute to building that consumer awareness.

The research office of Hawassa University will also benefit from the publications and can use the data for devising policy.

Application Submission:

Checklist for application submission:

☐Completion of required IRB training (http://compliance.vpr.okstate.edu/IRB/gs-CITI.aspx)
☐Grant Proposal, if research is externally funded
☐Outline or script of information to be provided prior to subjects' agreement to participate
☐Copies of flyers, announcements or other forms of recruitment
☐Informed consent/assent forms
☐Instrument(s) [questionnaire, survey, tests]
☐Resumes or CV's for all PIs (student or faculty) and advisors (4 page maximum for each)*

*CVs should highlight the education and research expertise of the researcher. Researchers may submit CVs prepared for federal grant proposals (e.g., NIH, NSF, USDA, etc.).

Appendices Included:

☐Appendix A - Request for OSU System Email Addresses for Human Subject Research Recruitment Purposes

Number of copies:

One (1), **single sided** paper copy of the application and associated attachments, signed by all PIs and advisor (if appropriate). Scanned/faxed signatures are acceptable.

Submission Address:

IRB/University Research Compliance
Oklahoma State University
219 Cordell North
Stillwater, OK 74078-1038

For assistance, please contact the IRB staff in the Office of University Research Compliance at 405-744-3377 or email irb@okstate.edu.
