# Supplementary material for: Iodine supplementation of lactating women and assessment of infant visual information processing and maternal and infant thyroid function: A randomized trial
Source: PLoS One. 2019 Oct 7;14(10):e0223348. doi: 10.1371/journal.pone.0223348 (PMC6779247; doi:10.1371/journal.pone.0223348)
Supplement: S1 Dataset — (PDF) [file pone.0223348.s003.pdf]

S1 Dataset. Data.

Group	Sub code	M_age	M_Wt0	M_Ht0	M_MUAC0	Goiter0	MUIC_0	M_Tg0
1	1	24		170.2	25.2	0	307.2	5.2
1	2	23		157	24	0	162.2	1.6
1	3	22		159.9	26	0	183.1	1.4
1	4	20	56	153.8	25.3	0	81.6	9.7
1	5	20	45	150.7	22.1	0	106.8	1.8
1	6	20	52.8	167.4	20.3	2	57.6	3.6
1	7	23	47	151.7	23.2	2	89.7	0.1
1	8	20	48.4	156.3	22.9	2	153.7	7.5
1	9	32	56.2	166.9	24.5	0	28.8	28.1
1	10	27	62.1	156.5	24.8	2	94.2	6
1	11	26	54.5	157	22	2	164.6	14.1
1	12	20	64.4	161.2	25	1	149.9	11.5
1	13	22	45.9	155	23.5	2	60.1	11
1	14	20	48.3	154.3	22.1	2	116.9	12.1
1	15	23	48.2	161.1	21.7	2	84	9.1
1	16	30	53.8	158.5	25.5	1	252.3	1.2
1	17	18	52.7	156.5	21.5	1	147.2	3
1	18	21	47.5	158.7	21.5	2	164.7	3.6
1	19	24	84.3	171.6	28.3	0	26.4	27.3
1	20	30	52.6	155.2	22.3	1	76	7.6
1	22	22	55	163.2	24.4	0	182.2	1.3
1	23	36	60	152	26.5	2	120.3	12.6
1	24	24	52.6	153.6	24.4	2	147.7	0.7
1	25	27	54.8	159.3	23	2	81.6	3.9
1	26	20	47.6	161.2	22	0	82.4	4.2
1	27	20	46.6	168.2	20.5	1	213.1	2.8
1	28	35	72.5	169.2	27	2	192.6	1.4
1	29	22	50.1	160.9	20.6	2	74.5	1.1
1	30	20	52.2	148.5	23	1	67	4.1
1	31	20	52.6	155.5	23.6	1	129.8	4.3
1	32	22	47.1	165.5	21	0	99.4	0.6
1	33	28	54.8	151	23.4	2	725.2	7.5
1	34	18	48.8	154.8	22.2	2	170.7	7.2
1	35	23	56.6	160	23.3	1	156.3	4.7
1	36	21	61	164.2	24.9	2	33.6	11.6
1	37	30	49.1	162.2	22.3	0	204.3	4.4
1	38	20	55	154.4	23.9	2	142.7	26.3
1	39	23	56	162.2	23.1	1	93.2	11.5
1	40	25	57.6	175.2	22.4	2	177.9	1.3
1	41	28	46.6	152.8	21.7	1	35.2	0.5
1	42	26	45.2	155.6	20.9	1	75.4	0.2
1	43	18	48.6	158.4	21.8	2	35.6	48.7
1	44	28	57.6	158.2	23.7	2	186.3	26.8
1	45	29	50.4	166.4	22	2	229.5	5.5
1	46	20	49.6	156.8	21.5	2	66.4	3.6

1	47	20	47.6	154.2	20.6	0	163.2	1.3
1	48	21	52.6	165.3	22	2	56.5	1.2
1	49	24	59.8	162.2	24	2	151.1	1.4
1	50	32	51.6	156	22.8	2	197	1.5
1	500	25	58.8	158.4	24.3	2	143.7	1.5
2	51	25	53	157.6	22	0	70.6	1.4
2	52	30	51.8	156.3	22.3	2	85.7	2.7
2	53	20	58.6	158.4	23.5	2	104.3	4.7
2	54	23	40	150.2	19.3	1	50.5	1.2
2	55	29	51	155.8	25	1	82.4	0.1
2	56	25	57	157	26	2	96	2.2
2	57	28	46	145	21.9	2	123.5	7.9
2	58	26		155.6	24.5	0	205.1	4.1
2	59	20	64.8	158.2	25.6	0	194.6	20.5
2	60	20	43.6	143.4	23	0	79.3	1.4
2	61	24	48.2	153	23.5	2	109.8	1.2
2	62	20		157.8	22.5	2	141.2	1.4
2	63	23	53.4	155.4	25	2	109.9	87
2	64	20	55.4	153.5	23.4	2	62.1	3.3
2	65	20	55.5	162.9	23	0	63.5	2.3
2	66	20	54.3	159.7	23	2	134.9	1.1
2	67	20	50.8	162.9	20.5	2	181.7	64.8
2	68	20	56.3	161.2	22.7	2	103.6	11.4
2	69	20	43.5	148.2	23.8	2	70.8	1.1
2	70	25	55	158.5	22.8	0	84	3.7
2	71	25	55	159.5	23.4	1	131.6	3
2	72	25	54.8	157.8	24	2	158.7	3.2
2	73	28	42.7	148	19.7	0	62.2	1.4
2	74	25	52.9	170	22.5	2	70.7	30.6
2	75	20	67.6	158.4	27.4	2	95	18.7
2	76	21	54.5	157.8	24.6	0	365.1	1.4
2	77	25	48.5	148.9	23.1	1	149.7	15
2	78	25	51.5	163	22.5	2	40.8	1.7
2	79	25	54.2	159.1	24.5	1	148.7	85.2
2	80	19	49.6	157	21	1	45.7	11.6
2	81	22	45.6	140.7	23.3	0	123.5	2.6
2	82	22	59	165	21.6	0	85	4.5
2	83	21	56.1	155.7	24	1	90	4.8
2	84	25	59.4	160.5	24.4	0	184	9.5
2	85	20	60.6	164.4	22.3	2	71.8	24
2	86	20	53.4	145.8	24	1	86	6.4
2	87	16	51.1	158.6	23.5	1	53	2.3
2	88	24	56.2	163	21.5	0	174.5	14.3
2	89	19		161.3	22.4	2	71.4	4
2	90	30		162.5	26.9	2	39.1	5.4
2	91	20	59	165.7	23.3	2	141.9	11.9
2	92	33	64.6	157.3	24.8	2	36.6	3.4

2	93	20	52.4	163.5	23.3	1	57.7	11.9
2	94	18	50.6	161	23	2	301.9	9.5
2	95	20	64.4	160.8	23.5	2	160	311.7
2	96	22	53.8	149.8	23.8	2	62.5	24.1
2	97	19	59.2	157.5	26.4	0	169.4	4
2	98	18	57.4	160.6	24	2	116.2	34.3
2	99	21	43.6	148.3	24.8	2	34.7	7.2
2	100	23	47.6	152	19.7	1	137.7	3.6
2	501	19	58.8	158.6	23.9	1	46.5	6.4

M_T30	M_T40	M_TSH0	BMIC_0	Child_age	C_Wt0	C_length0	UIC_C0	Mgoiter6
0.76	3.6	5.28	157.27	9	4.5	54	265.7	0
1.45	6.71	6.09	36.75	2	2.6	44.5	130.8	0
1.26	6.3	2.59	495.73	4	3.3	48	292.9	0
1.46	5.95	3.96	11.26	3	2.9	49.5	417.4	0
1.05	4.61	6.29	18.23	10	3.5	50.1	85.3	0
2.06	7.76	1.58	10.8	4	2.4	48	400.6	0
1.28	10.35	5.28	20.36	9	3.5	52	89	0
1.03	6.47	2.92	15.68	9	3.8	53	310	0
1.28	5.89	5.55	7.78	8	2.7	53	256.1	0
0.79	4.29	0.07	36.65	6	4.9	55	141.4	0
0.88	5.24	4.35	10.27	5	3.2	48	221	1
1.34	5.99	1.74	24.91	8	3.5	48	85.8	1
1.14	5.14	0.51	8.11	6	3.8	52.5	86.7	1
1.33	8.08	4.87	74.98	3	2.9	49.5	616.1	1
1.87	9.12	1.36	19.09	3	3.6	51	169.1	1
0.57	6.04	5.99	146.34	8	4.3	53	110.8	0
1.97	9.85	6.35	344.93	6	3.1	47	307.7	0
0.99	7.22	1.05	192.58	6	3.2	52.2	372.2	1
1.03	7.7	1.92	130.57	6	3.8	54.7	92.4	0
1.04	8.6	0.23	117.07	5	3.9	51	131.6	0
1.07	6.38	3.03	263.89	8	3.2	52.5	397.5	0
1.28	10.72	1.43	131.76	3	3.2	48.5	1037	1
2.81	6.63	2.7	199.91	7	2.9	49	172.3	1
1.68	7.22	3.36	334.79	3	3.4	50.5	326.4	0
1.83	7.69	11.46	272.07	2	2.9	46	1113	0
1.28	7.81	7.49	111.03	3	2.3	45	106	1
1.19	6.97	1.09	203.76	5	3.6	50.7	218.7	0
1.07	10.87	3.79	150.78	5	2.5	49.8	59.3	1
1.3	22.2	3.71	81.54	4	3.2	49.6	138.2	0
1.47	10.12	0.35	219.18	7	3.5	50	285.8	1
2.38	6.54	4.66	69.83	5	3.7	51	68.9	0
2.04	6.57	4.24	331.42	3	2.6	45.8	2878.9	0
1.35	8.14	0.66	164.98	4	3.7	52.5	159.9	1
1	7.17	1.92	164.98	3	2.9	49.9	299	0
1.13	7.85	0.78	264.38	8	4	53.5	417.8	1
1.2	7.05	0.07	283.15	6	3.5	51	218.2	0
1.26	8.35	1.05	476.46	4	3	48.5	497	0
2.09	12.28	0.07	146.99	4	2.9	46	103.1	0
1.92	9.62	1.05	174.75	5	3.1	50	906.1	0
1.03	5.24	1.2	137.85	8	3.5	50.5	246	0
0.68	7.63	1.2	467.65	9	2	44.8	449.7	0
0.82	5.65	4.17	105.93	3	2.9	48.5	102.4	1
1.21	7.16	1.13	422.58	4	3	51	284	0
1.34	6.37	0.51	474.87	3	2.6	47	336.9	0
1.69	5.42	6.19	158.88	4	2	46	87.3	0

1.62	5.99	3.43	472.74	6	3.4	50.5	1203.5	0
0.75	5.06	1.2	49.5	9	4.3	56.5	123.9	0
0.78	6.98	0.47	163.95	5	3.1	47.8	194.9	0
1.77	4.93	2.89	421.4	2	3.4	48.5	182.4	1
1.7	5.86	3.61	97.59	2	2.9	46.4	368.4	0
1.72	6.09	1.89	158.62	7	3.1	49.7	160.3	0
1.3	6.3	3.21	445.4	2	3	50.5	103.9	0
1.16	5.19	6.55	163.12	6	3.1	51.3	290.9	0
2.25	7.69	0.43	101.04	9	2.6	47	43.1	0
1.13	5.33	1.55	143.7	9	3.8	53	109.1	1
1.21	5.73	1.85	185.53	8	4	51	106.4	1
1.52	5.5	1.55	380.7	7	2.4	44.4	302.5	1
0.76	4.3	3.28	94.02	8	4.9	57.5	111.1	0
1.41	6.91	0.27	255.52	7	3.1	51	289.5	0
1.39	9.75	3.71	219.86	7	2.1	44.5	160	0
1.16	5.85	0.55	153.78	7	3.3	54	238.6	0
0.82	4.72	0.58	147.58	8	3.5	52	156	1
1.17	5.14	0.28	337.85	9	3.7	50.5	39	1
1.74	7.23	0.86	242.57	4	2.2	47	363.6	1
1.83	7.09	1.81	225.32	2	2.5	46	331.4	0
1.18	8.37	0.78	90.64	9	3.5	52	65.1	1
1.21	6.39	0.26	163.65	6	4.3	52.5	262.2	1
1.25	5.27	3.03	128.28	5	3.3	49.5	65.9	1
1.26	5.2	0.93	123.95	9	3.6	52	107.2	1
1.22	6.83	1.05	118.47	5	3.7	54	115	0
1.29	6.55	3.03	200.25	4	3.8	51.5	393.6	1
1.99	8.24	3.68	218.22	5	2.6	46.5	316.3	1
1.48	5.43	2.74	198.35	7	3.2	51.3	295.4	0
1.33	7.69	0.7	9.56	10	3.5	52	107.6	1
0.77	5.38	0.31	8.54	10	3.8	52.5	95.3	1
1.38	7.96	0.86	28.46	6	2.8	49.2	687.9	0
1.62	7.83	0.26	21.16	7	3.3	50.5	435.8	0
1.96	7.57	0.39	19.31	3	3.4	49	206.7	0
1.83	6.49	0.03	82.43	4	1.5	41.5	464	0
1.48	6.46	2.92	16.5	5	3.2	51	89.4	0
1.03	4.89	4.52	22.5	3	3.5	52.1	347.7	0
0.68	5.15	3.86	19.35	5	3.3	47.8	75.5	0
1.52	5.13	3.1	18.9	7	4	50.4	143.2	0
1.23	6.89	3.63	9.03	5	4.3	54.3	53.9	0
1.08	11.79	1.66	12.15	8	3.5	51.6	119.1	0
2.27	8.9	2.1	28.18	6	3.5	49	173.6	0
1.33	8.34	2.3	11.55	8	3.2	51.3	94.8	1
1.72	8.96	5.96	110.85	2	2.2	47	201.8	0
1.89	6.79	3.78	528.79	5	2.2	45.5	449.5	0
1.54	7.85	1.34	156.48	6	4.4	52.8	173.9	1
1.42	7.2	2.83	200.37	6	2.4	45.5	174.4	1
1.45	10.12	1.13	248.67	7	4.4	50.8	222.1	1

1.64	12.14	2.6	54.7	8	3.3	50.5	103.4	0
1.54	9.03	2.53	965.28	5	2.6	46.5	685.2	0
1.95	5.91	0.22	322.39	7	2.8	46.7	961.6	2
1.5	7.2	3.76	291.18	4	3.3	49	498.5	1
1.62	6.54	2.75	193.37	7	2.9	48.5	247	0
2.24	9.37	3.63	331	6	3.1	50.3	695.7	1
1.97	4.12	1.66	327.18	2	2.8	47.2	193.3	1
1.39	6.38	0.85	356.06	9	3.3	52	280	0
2.37	10.54	4.76	195.97	3	3.2	48.5	487.7	0

MUIC_6	MTg6	MT3_6	MT4_6	MTSH_6	BMIC_6	C_sex	C_Wt6	C_length6
369.1	8.5	0.68	5.19	3.15	429.92	2	9.06	67
205.4	0.8	0.9	3.74	3.68	134.32	1	6.9	62.2
86.3	2.1	0.59	5.2	1.97	195.1	2	7.56	64.4
94.3	4.2	1.2	6.73	1.7	43.48	1	7.78	63.9
241.3	3.1	0.93	5.57	3.31	201.3	1	8.08	68
91.1	4.1	1.07	6.91	1.68	54	1	6.14	62.4
153.9	3	1.51	9.48	2.77	18.55	1	7.92	64.5
293.2	4.4	1.09	6.66	1.61	19.79	2	7.66	65.6
44.2	26.6	0.34	6.47	1.83	17.24	2	7.7	64.3
250.4	1.5	0.67	5.88	1	29.47	1	11.38	70.3
204.2	6.4	0.84	4.65	2.64	40.78	1	6.12	61.4
154	48	1.09	6.64	0.24	10.44	2	8.06	63
107.5	7.3	1.3	5.63	0.85	8.65	1	8.5	66.8
436.9	25.6	1.03	8.24	1.15	40.9	1	6.86	66.7
62	10.4	1.33	7.56	0.88	34.3	1	7.14	63.7
275.4	1.6	1.27	5.75	2.83	32.65	2	6.42	66
182.1	3.2	1.15	7.25	2.38	7.79	2	5.66	58.4
38.6	2.6	1.09	5.75	0.93	9.22	1	7.96	66.3
83	22.7	1.76	7.11	0.21	14.04	2	8.48	68.5
115.9	10.7	1.21	4.76	0.3	20.73	2	7.58	63.2
235.7	1.8	0.75	8.39	3.78	261.65	2	6.4	64.9
153.4	3.1	1.12	6.47	0.36	138.25	1	7.58	63.5
130.2	3.9	1.21	9.88	2.75	178.25	2	5.46	59.6
146	2.3	0.48	7.76	2.06	156.03	1	8.16	66.5
45.9	0.7	0.72	7.89	2.34	226.59	1	6.78	63.5
338.3	1.5	1.12	4.63	6.24	111.64	2	5.36	58.2
65.9	1.4	1.69	6.32	0.3	63.84	2	7.92	65.2
66.9	3	1.6	7.67	2.1	82.86	2	6.5	61
104.1	5.2	0.89		3.46	53.1	2	6.52	63.8
135.1	7	1.65	6.23	0.9	54.37	2	8.04	64.1
196.8	5.9	1.71	8.81	3.66	105.11	2	6.46	66
33.3	13.8	0.86	12.08	2.68	83.7	1	7.1	58.9
111.3	5.5		7.42	1.15	276.54	2	7.82	63
191.4	4.1	1.22	7.96	1	145.77	1	5.4	61.7
80.6	5.4	1.43	8	0.75	228.93	1	7.16	67.3
118	13.9	1.26	10.22	1.17	152.13	2	6.38	63.1
402.3	45.4	1.2	11.17	0.98	381.87	2	7.1	61.9
518.4	4.8	1.43	9.48	0.36	214.94	1	7.42	62.5
215.1	1.3	1.65	9.54	0.95	140.06	1	8.12	68.3
200.3	1.5	1.15	7.59	1.32	102.92	1	7.38	66.5
56.2	0.7	1.15	5.64	1.63	44.19	1	6.84	61.7
300.2	10.4	0.96	5.25	1.25	957.73	2	5.52	62.1
125.5	26.9	1.4	6.68	0.24	163.62	2	7.34	66.8
162.3	5.8	0.58	4.24	1.1	112.65	1	6.68	60.9
210.6	3	1.2	5.35	1.39	316.38	2	5.22	57.1

192.6	1.6	1.44	5.8	0.5	249.55	1	5.02	58.1
75.7	0.7	1.18	4.83	1.05	41.93	2	7.82	68.6
96.6	14.9	2.2	8.12	0.22	166.59	2	6.44	62
70.7	4.2	0.87	4.96	1.13	98.86	2	7.88	63.7
448.9	0.6	0.87	4.98	0.61	643.05	1	7.86	65
92.2	2.7	1.11	5.03	2.73	138.85	1	7	63
47.2	4.9	1.26	5.91	2.34	79.83	1	7.32	66.9
108.8	4.6	1.14	6.01	2.57	68.5	2	7.42	65.7
644.9	0.9	1.56	6.64	1.52	385.1	1	6.08	63
126.3	1.9	1.16	4.95	0.59	152.43	1	8.2	69
162.7	3.4	1.25	5.75	0.69	38.19	1	7.3	65.3
85.7	10.3	0.73	4.16	1.95	116.96	2	6.44	60.5
354.7	6.9	1.51	4.54	1.26	133.12	2	6.64	65.7
162.5	9.9	1.45	5.15	1.23	77.48	2	6.46	64
274.5	1.1	1.29	4.75	1.83	362.28	2	5.1	61.3
294.4	0	1.3	5.06	0.92	257.57	1	7.1	67.7
203.9	4.3	1.92	5.56	0.41	234.3	2	6.6	63
189.2	36.5	0.77	5.38	1.08	77.42	2	7.22	63.2
174	3.5	1.41	5.49	1.1	316.83	2	5.16	58.5
46.5	1.8	1.5	5.49	1.65	189.01	2	7.2	63
90	7.2	2.16	9.47	1.15	212.5	2	6.32	60.8
228.6	20.3	1.33	7.04	0.81	156.68	1	7.5	62.6
234.1	8.1	0.87	5.16	2.05	129.28	1	7.7	64.7
190.7	4.9	1.36	7.69	0.2	76.5	2	5.58	64
129.6	2.9	1.55	9.57	0.44	47.07	1	7.82	67.5
428	7.8	0.88	5.45	2.11	145.5	1	7.5	65.2
87.4	4.2	0.75	4.85	2.07	335.26	1	6.36	60.4
50	4.7	2.11	7.21	1.82	180.38	1	7.98	65.2
295.2	28.8	1.79	7.77	1.02	335.63	2	7.14	64.7
77.4	15.4	0.99	6.85	0.68	53.24	2	7.32	62
68.1	0.9	1.73	5.21	1.28	111.13	1	6.1	62.6
604.4	12.1	1.62	8.96	0.46	178.76	1	7.04	62.8
92.2	5.4	1.53	7.51	1.18	143.1	1	7.5	65.5
45.5	151.7	1.21	4.34	0.08	245.18	2	6.5	58.3
84.6	4.8	1.14	5.63	1.71	278.21	1	6.7	64.3
100	0.7	0.62	2.78	4.01	7.85	1	9.32	68
73	2.2	0.52	3.31	1.56	10.52	2	8.38	64.6
216.2	3.7	0.52	3.83	2.44	13.4	1	9.48	66.1
94.2	2.2	0.91	5.36	2.74	18.14	1	7.32	66.8
190.7	18.7	0.77	6.96	0.71	19.31	2	6.32	61
110	2.1	1.36	5.39	0.77	15.44	2	6.46	62.5
353.8	0.9	1.2	5.17	2.83	34	2	6.74	63.3
61	8.1	1.19	4.36	1.11	53.32	1	6.24	61.5
157.7	2.2	1.02	3.52	0.66	12.75	2	6.52	59.5
51.9	2.8	0.97	4.31	0.33	10.47	1	9.36	66.5
47	3.6	1.4	6.03	1.41	3.68	2	7.58	58.2
162.9	1	0.96	8.07	0.53	17	2	8.34	61.5

72.4	6.9	1.2	5.11	1	15.61	2	7.44	62.5
158.3	3.5	1.01	7.22	0.85	15.03	2	8.32	62.7
85.4	121.2	1.45	3.85	0.09	89.05	2	6.82	63.2
56.7	7.6	1.33	3.88	0.69	71.2	1	7.54	63.5
79.8	1.5	1.21	5.7	1.75	98.29	2	7.56	64.5
29.6	74.2	2.05	8.8	1.07	134.48	1	6.18	62.6
138.7	3.2	1.59	5.08	0.02	208.68	1	7.14	63.2
38	1.3	0.63	3.42	0.57	228.78	1	6.54	61.8
191.1	1.7	1.32	5.89	1.13	202.38	1	7.16	61.5

CUIC_6	C_TSH6	C_T4_6	HFIAS	Tot_Nov_Q	Tot_shift	Num_looks	Ave_look
535.9	1.14	12.95	1	0.59	5	7	3.93
292.9	0.1	7.45	2	0.72	6	10	11.14
374.1	0.75	11.08	1	0.49	5	4	13.1
244.6	1.01	8.47	1	0.41	5	4	17.85
309.4	0.36	8.09	1	0.47	1	5	10.52
103.3	0.36	11.27	3	0.63	2	6	11.48
312.1	0.1	19.9	3	0.88	3	9	6.87
120.7	0.62	18.35	3	0.29	5	8	5.56
257.2	0.1	17.81	4	0.45	3	5	2.66
615.7	0.1	14.31	3	0.34	5	6	5.94
384.3	0.62	10.31	4	0.32	6	8	3.74
151.9	2.95	15.11	1	0.34	3	6	5.91
305.8	1.66	7.61	1	0.63	5	6	10.66
734.4	2.18	14.4	3	0.35	9	7	17.33
364	2.82	7.94	1	0.36	4	6	6.77
616	1.66	18.6	2	0.76	5	5	9.08
101.5	2.31	19.9	1	0.24	3	7	9.64
144.1	0.1	9.9	2	0.54	2	4	6.13
105.8	0.75	10.39	1	0.41	1	9	6.28
271	0.23	9.06	1	0.41	6	7	3.04
172.8	2.95	13.35	2	0.5	0	4	16.53
492.8	1.27	9.2	1	0.54	7	5	8.01
610.9	0.1	8.02	4	0.74	3	7	13.96
133	0.1	8.47	1	0.49	5	6	12.37
236.6	0.1	8.18	3	0.56	10	5	10.12
103.3	1.66	16.16	1	0.43	10	8	6.52
72.4	0.1	19.8	1	0.18	1	5	26.03
81.4	1.01	9.37	1	0.16	2	5	8.92
65.6	0.1	8.84	1	0.26	1	7	9.86
63.3	0.62	9.88	3	0.5	4	7	19.09
143.4	1.79	11.2	1	0.34	3	7	25.67
284.6	0.36	7.91	1	0.63	6	9	4.34
193	0.23	12.33	1	0.47	8	6	14.63
105.1	0.1	13.39	1	0.46	4	4	5.23
272.7	1.01	11.43	1	0.45	9	5	7.51
451.8	0.49	10.66	1	0.45	3	13	3.01
650.2	2.05	9.15	1	0.37	2	4	14.09
544.9	0.1	7.61	1	1	0	4	11.48
250.1	0.23	10.35	3	0.3	7	8	5.84
221	2.82	11.1	4	0.48	2	4	21.54
103.1	0.1	13.28	1	0.65	3	5	7.35
453.9	0.36	10.89	1	0.47	9	4	2.25
183.5	0.88	20.2	1	0.66	8	6	9.26
164.8	1.14	15.89	3	0.52	2	5	2.42
391.9	0.23	10.57	3	0.43	4	6	16.66

423.1	0.1	7.88	3	0.52	5	6	9.46
114.2	0.23	8.48	1	0.36	5	8	2.1
271	2.05	11.1	2	0.59	1	6	2.89
174.6	0.1	13.31	3	0.18	4	5	8.48
584.2	4.23	10.26	3	0.35	4	5	6.06
175.5	0.1	7.8	1	0.47	8	4	8.16
273.4	9.9	21.12	1	0.47	8	6	2.97
94.8	2.05	10.28	4	0.05	1	4	19.54
436.1	0.88	8.18	2	0.77	1	6	3.38
145.3	0.75	10.2	4	0.5	0	6	4.72
39.8	0.1	20.7	1	0.39	5	4	4.94
170.2	0.1	10.63	3	0.66	4	7	17.17
136.2	1.66	12.36	2	0.39	1	7	6.71
69.8	0.1	8.42	1	0.48	7	10	5.05
869.2	0.62	11.67	3	0.55	6	4	7.17
250.8	0.88	20.5	2	0.7	2	6	9.68
233.1	1.14	12.06	1	0.76	4	8	4.91
356.2	0.49	11.64	3	0.36	6	5	6.64
275.7	1.66	7.8	1	0.45	6	10	2.6
82.5	0.1	10.89	1	0.45	4	6	13.73
571.4	0.1	9.57	1	0.74	2	4	9.09
398.7	1.4	9.55	1	0.33	7	9	6.79
221.8	3.46	27.4	3	0.17	4	6	10.28
282.2	0.23	14.36	1	0.5	0	7	4.01
47.6	0.1	14.5	3	0.52	7	4	8.89
439.7	1.14	17.6	4	0.73	2	9	9
851.5	0.1	9.53	1	0.56	5	7	8.57
806.7	0.49	12.03	1	0.31	5	6	11.55
208.8	0.88	15.83	3	0.74	8	8	5.9
169.3	0.49	9.29	1	0.47	6	15	4.97
138.4	1.53	14.04	1	0.64	1	4	9.57
297.5	0.88	20.7	1	0.72	4	7	9.31
218.1	0.1	7.07	2	0.56	7	7	23.08
806.7	0.12	14.33	1	0.09	1	8	2.58
850.1	0.89	19.31	1	0.83	4	16	5.91
68.4	0.12	16.65	4	0.65	4	4	4.03
38.5	0.13	10.13	1	0.6	5	7	5
188.4	6.08	17.31	2	0.65	4	8	9.99
351.6	0.12	15.51	1	0.66	3	6	5.91
148.6	0.7	12.77	1	0.89	3	10	4.24
129.2	17.95	15.4	1	0.38	6	7	7.03
146	0.7	13.95	4	0.87	1	6	25.37
78.6	0.14	17.13	1	0.58	5	6	6.51
524.2	0.51	20.77	1	0.66	5	22	1.63
108.4	1.36	26.06	1	0.27	8	4	10.78
63.1	0.13	11.53	1	0.46	2	5	5.61
84.8	0.13	11.79	1	0.47	7	5	4.54

252.5	0.13	10.56	1	0.34	4	12	5.8
360.9	1.55	19.63	2	0.67	10	7	5.98
82.3	7.6	24.84	1	0.37	5	12	2.31
194.5	0.13	12.68	1	0.76	5	4	3.71
113.9	0.13	23.28	4	0.41	3	4	5.7
59.7	0.33	16.2	2	0.6	4	6	9.02
299.5	0.13	12.11	4	0.96	1	11	14.83
245.1	0.89	18	3	0.58	4	6	4.87
161.4	1.27	15.71	1	0.38	7	7	20.06

Long_look	W_edu
9.97	2
34.02	4
36.54	3
31.97	2
19.96	4
37.24	4
18.09	2
9.56	2
5.43	1
11.1	1
9.5	2
11.07	2
29.1	2
30.68	6
12.48	1
19.3	1
19.98	3
16.19	3
14.34	3
5.03	1
41.5	2
15.29	1
35.56	2
34.82	3
30.08	3
9.25	2
107.8	1
19.24	3
27.82	3
44.59	5
54.14	1
8.27	3
34.93	4
13.21	2
15.21	2
8.13	1
22.09	3
20.99	2
15.96	1
55.89	1
14.5	6
4.18	3
18.87	2
5.02	3
30.75	1

20.34	1
3.69	5
5.48	3
17.34	1
11	1
17.14	1
4.91	1
61.63	1
6.63	1
8.41	1
12.83	1
57.38	2
19.43	1
9.83	1
12.78	1
20.25	1
11.57	2
14.97	6
4.1	3
19.59	2
20.89	2
12.67	4
18.79	1
7.43	2
22.68	1
16.03	2
22.92	1
19.02	2
11.84	2
10.46	6
21.52	3
17.66	3
73.93	1
6.35	1
11.37	6
8.11	1
9.31	4
19.73	2
12.98	4
8.04	3
17.95	3
105.02	3
11.38	1
2.97	3
34.69	1
17.95	1
11.18	1

11.12	6
12.18	1
3.52	3
8.66	6
9.55	3
19.57	4
43.73	1
8.02	1
46.02	6
